# Bioactive Profile, Antioxidant, and Antimicrobial Activity of Sweet and Hot Peppers at Different Stages of Ripeness

**DOI:** 10.3390/antiox15060756

**Published:** 2026-06-15

**Authors:** Elena Coyago-Cruz, Gabriela Méndez, Santiago Buyancela, Fátima Rodríguez-Muñoz, Jorge Heredia-Moya

**Affiliations:** 1 Carrera de Ingeniería en Biotecnología, Universidad Politécnica Salesiana, Sede Quito, Campus El Girón, Av. 12 de Octubre N2422 y Wilson, Quito 170109, Ecuador; 2 Centro de Investigación Biomédica (CENBIO), Facultad de Ciencias de la Salud Eugenio Espejo, Universidad UTE, Quito 170527, Ecuador

**Keywords:** functional foods, antibacterial activity, antifungal activity, capsaicin

## Abstract

The genus *Capsicum* is widely used worldwide for its culinary value and functional potential. The objective of this study was to evaluate the bioactive compounds, antioxidant and antimicrobial activity of sweet and hot pepper at different stages of ripeness. Six varieties of peppers at five stages of ripeness were analysed. Mineral parameters (Ca, Fe, Na, K, Mg) were determined by atomic absorption spectrophotometry, while bioactive compounds (vitamin C, organic acids, carotenoids, and phenols) were analysed by liquid chromatography. Antioxidant activity was evaluated using ABTS and DPPH assays, and antimicrobial activity was assessed by minimum inhibitory concentration against bacteria and yeasts. Multivariate analyses (PCA and heatmap) were carried out at a significance level of *p* < 0.05. The results showed that genotype was the main determinant of variability, surpassing the effect of ripeness. Potassium was the predominant mineral (3431.5 mg/100 g DW) in Malagueta chilli M5. Variety-specific peaks were identified, notably vitamin C in Habanero chilli (M3) (10,319.5 mg/100 g DW), capsaicin in Malagueta chilli (M5) (1949.8 mg/100 g DW), and carotenoids in Orange medium peppers (M5) (9495.8 mg/100 g DW). Antioxidant activity was higher in hot varieties (41.3 mmol ET/100 g DW in Habanero chilli (M2) by DPPH), while antimicrobial activity varied against *Escherichia coli* (2.6 mg/mL in Yellow medium peppers (M4)), *Staphylococcus aureus* (5.2 mg/mL in Orange medium pepper), and *Streptococcus mutans* (2.0 mg/mL in Jalapeño chilli), with low MIC values. Multivariate analyses confirmed that chemical and biological variability is primarily structured by genotype.

## 1. Introduction

The genus *Capsicum*, belonging to the Solanaceae family, is one of the most important horticultural groups worldwide in agriculture, economics, and nutrition, due to its wide distribution, genetic diversity, and versatility in human diets [1,2,3]. This genus is native to the tropical and subtropical regions of South America, where it was domesticated. Currently, the genus *Capsicum* is recognised as comprising 20–38 species, of which five have been fully domesticated and cultivated, such as *Capsicum annuum*, *C. frutescens*, *C. chinense*, *C. baccatum*, and *C. pubescens*. The domestication of these species occurred in different geographical regions, with *C. annuum* initially domesticated in Mexico, *C. frutescens* in the Caribbean, *C. chinense* in the Amazon basin, and *C. baccatum* and *C. pubescens* in Bolivia [4].

All cultivated *Capsicum* species exhibit this morphological variability, with bell peppers being among the most representative varieties of *C. annuum*, widely cultivated for their mild flavour, characteristic aroma, and colour diversity. During ripening, the fruits undergo a colour transition from green to mature shades such as yellow, orange, red, dark red, and purple, which is closely related to metabolic and biochemical changes in the fruit [5].

Within the genus *Capsicum*, *Capsicum annuum* is the most widely distributed and cultivated globally, available in multiple shapes and sizes and in both sweet and spicy varieties, including bell peppers, New Mexico chillies, and cayenne peppers, among others [6]. The fruits can be used as a vegetable when unripe, as a spice when dried and ripe, and as a raw material for processed products such as sauces, pickles, and condiments, which are marketed globally [7]. Additionally, some *Capsicum* varieties are cultivated for ornamental purposes due to their compact growth and the diversity of fruit colours [8]. In Ecuador, according to FAO statistics for 2023, peppers and green chillies ranked 35th among 55 reported agricultural products, underscoring their importance to the country’s production [9].

From a chemical and functional point of view, *Capsicum* fruits are recognised as an important source of bioactive compounds, including carotenoids, phenolic compounds, antioxidant vitamins and capsaicinoids. The characteristic colour of ripe fruits is attributed to the combined effect of pigments such as capsanthin, capsorubin, cryptoxanthin, zeaxanthin, and other carotenoids, whose concentration increases as the ripening process progresses [6]. In turn, up to 50 phenolic compounds have been identified in *Capsicum annuum*, including 21 flavonoids and 20 phenolic acids, as well as lignans and stilbenes, whose distribution depends on the ripeness and colour of the fruit [10,11,12].

Recent studies have reported the presence of at least 12 different bioactive compounds in sweet pepper fruits, such as quercetin and its derivatives, *L*-tryptophan, phytosphingosine, ginger glycolipid A, tetrahydro-pentoxylin, blumenol C glycoside, cholecenic acid, and capsoside A, with significant variations in their abundance between unripe green fruits and ripe red fruits [12]. These metabolites have been associated with multiple biological effects, including antioxidant, anti-inflammatory, antimicrobial, anticancer, antidiabetic, antiadipogenic, and neuroprotective activities, mainly due to their ability to neutralise free radicals and reactive oxygen species [13,14].

Likewise, *Capsicum annuum* has been identified as a significant source of natural antioxidants due to its high content of polyphenols, carotenoids, vitamin C, vitamin E, and capsaicinoids. Among these, capsaicin stands out for its anti-inflammatory, antiproliferative, and analgesic actions. This molecule is used in topical formulations for pain treatment and has been shown to significantly reduce inflammation [12]. Furthermore, extracts of *C. annuum* have been observed to reduce intracellular reactive oxygen species levels, thereby reinforcing the body’s antioxidant mechanisms [12,15]. In turn, the flavonoids present, such as quercetin, luteolin and apigenin, play a key role in antioxidant and anti-inflammatory effects [16,17].

Beyond their antioxidant capacity, *Capsicum annuum* extracts have been shown in various studies to exhibit antimicrobial and antifungal activity against pathogenic microorganisms such as *Escherichia coli* and *Staphylococcus aureus* [18]. Aqueous extracts of green, red, and yellow peppers also exhibited antimicrobial activities [19]. In addition, capsaicinoids, especially capsaicin, have been extensively studied for their antimicrobial action against pathogenic bacteria and fungi, reinforcing the functional potential of these fruits [18,20].

Although several studies have investigated specific groups of bioactive compounds or antioxidant properties in *Capsicum* fruits, most have focused on individual cultivars, limited ripening stages, or a restricted set of metabolites. To the best of our knowledge, no previous study has simultaneously integrated physicochemical traits, mineral composition, vitamin C, organic acids, anthocyanins, carotenoids, phenolic compounds, antioxidant capacity, antimicrobial activity, and multivariate analyses across both sweet and hot pepper genotypes at five ripening stages under tropical Ecuadorian conditions. Therefore, this study provides a comprehensive evaluation of the biochemical and biological changes associated with fruit ripening and genotype diversity. In this context, the objective of this study was to evaluate the bioactive compounds and antioxidant and antimicrobial activity of sweet and hot peppers at different stages of ripeness. The results will provide insight into the impact of ripening on the bioactivity of Capsicum annuum, offering relevant scientific evidence for its assessment as a source of natural functional ingredients with potential applications in the food, nutraceutical, and pharmaceutical industries.

## 2. Materials and Methods

### 2.1. Physicochemical Analyses

**Figure 1:**
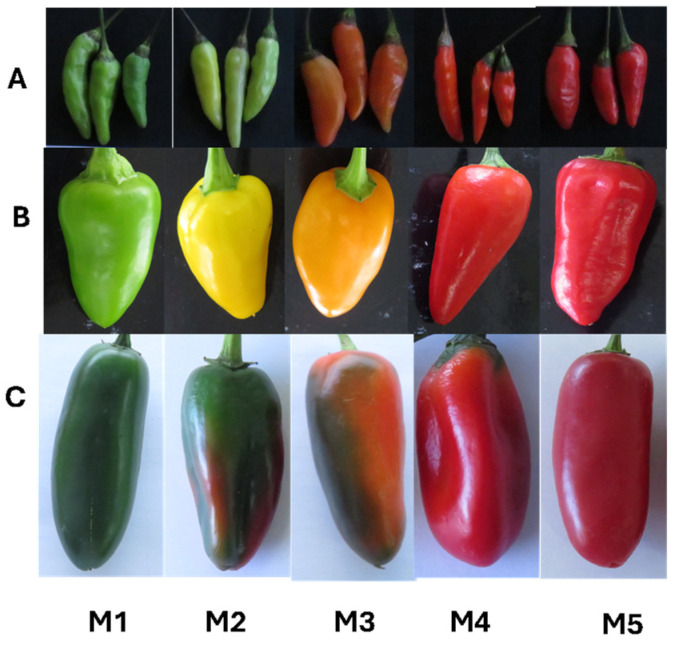
Photograph of three varieties of chilli peppers at five stages of ripeness. Note: (**A**) Malagueta chilli (*Capsicum frutenses* L.); (**B**) Habanero chilli (*Capsicum chinense* Jacq.); (**C**) Jalapeño chilli (*Capsicum annuum* L.); M1, maturity at 0%; M2, maturity at 10%; M3, maturity at 50%; M4, maturity at 80%; M5, maturity at 100%.

**Figure 2:**
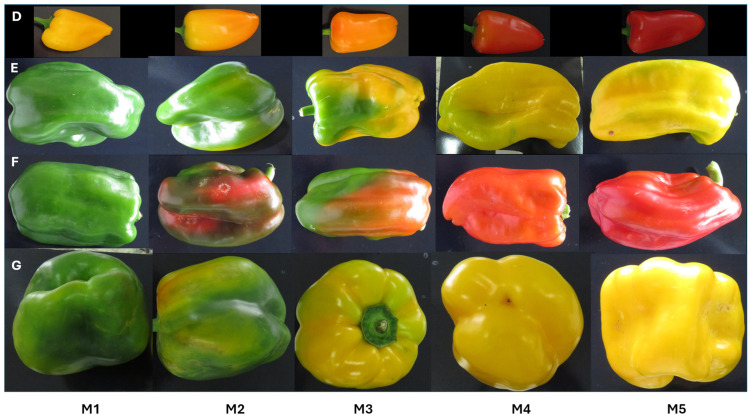
Photograph of four varieties of sweet pepper (*Capsicum annuum* L.) at five stages of ripeness. Note: (**D**) Orange cherry peppers; (**E**) Yellow medium peppers; (**F**) Orange medium peppers; (**G**) Yellow big peppers; M1, maturity at 0%; M2, maturity at 10%; M3, maturity at 50%; M4, maturity at 80%; M5, maturity at 100%.

The plant material was selected from local plantations in Ecuador using a random sample of 2 kg of fruit from the same crop [21]. The samples consisted of three varieties of hot peppers (Malagueta chilli, Habanero chilli, and Jalapeño chilli) (Figure 1) and four of sweet pepper (Orange cherry peppers, Yellow medium peppers, Orange medium peppers, and Yellow peppers) (Figure 2). The samples were selected based on the fruit’s external colour at five stages of ripeness (M1 through M5), with M1 being the least ripe and M5 the most ripe. The sample was divided into two fractions. In the first fraction, which consisted of fresh material, physicochemical properties were quantified, such as weight (g), longitudinal diameter (mm), equatorial diameter (mm), pH, soluble solids (°Brix), titratable acidity (%) by titration with 0.1 M sodium hydroxide, ash content (%) at 550 °C in a muffle furnace (Thermo Fisher Scientific, Waltham, MA, USA), and moisture content (%) at 110 °C using a Be20 oven (Memmert GmbH Co. KG, Schwabach, Germany). The second fraction was cut into small pieces, the seeds were removed, it was frozen, and it was freeze-dried in a Christ Alpha 1-4 LDplus (Martin Gefriertrocknungsanlagen GmbH, Osterode am Harz, Germany). The freeze-dried sample was ground into a fine powder and stored in amber glass vials until analysis [22].

#### Mineral Analyses

For mineral analysis, 40 mg of freeze-dried powder was weighed into a Teflon digestion vessel (Berghof Products + Instruments GmbH, Eningen unter Achalm, Germany), and 5 mL of 65% HNO_3_ was added. The reaction was allowed to stand for 10 min. The sample was digested in a Speed-180 Xpert microwave oven (Berghof Products + Instruments GmbH, Eningen unter Achalm, Germany) at a gradient of 140 °C, 30 bar, 70% power for 5 min; 200 °C, 35 bar, 80% power for 15 min; 50 °C, 25 bar, 0% power for 10 min. The extracted sample was made up to 25 mL with deionised water. Mineral determination was performed on a Varian SpectrAA-555 atomic absorption spectrophotometer (Varian Inc., Palo Alto, CA, USA) using specific lamps for iron (372.0 nm and 0.20 nm slit), potassium (404.4 nm, 0.5 nm slit), magnesium (202.6 nm and 1.0 nm slit), and sodium (589.6 nm, 0.5 nm slit). The calibration curve was prepared using standard solutions with a concentration of 1000 ppm. Each sample was analysed in triplicate, and readings were taken in duplicate. The concentration of each mineral was expressed as milligrams of the mineral per 100 g of freeze-dried sample (mg/100 g DW) [23].

### 2.2. Analysis of Bioactive Compounds

The total anthocyanin content was determined using the pH differential method. Ethanolic extracts were prepared from 20 mg of freeze-dried material using 2 mL of 96% ethanol, followed by 3 min of sonication and filtration through a 0.45 µm PVDF filter. For analysis, 50 µL aliquots of the extract were placed in a microplate, with 200 µL of potassium chloride buffer solution (0.025 M, pH 1.0) added to one well and 200 µL of sodium acetate buffer solution (0.4 M, pH 4.5) added to another. Absorbances were recorded at 520 and 700 nm using a BioTek microplate reader (Agilent Scientific Instruments, Santa Clara, CA, USA). Quantification was performed using a calibration curve with cyanidin-3-glucoside, with results expressed in dry weight equivalents.

For the general extraction of bioactive compounds, which were quantified by high-performance liquid chromatography, 20 mg of lyophilised material was used and extracted with specific solvents depending on the metabolite group. The samples were homogenised and treated in an FS60 ultrasonic bath (Fisher Scientific Inc., Waltham, MA, USA) to promote compound release, with processing times adjusted to the analytical method. Subsequently, they were centrifuged at 14,000 rpm for 4 min in an Eppendorf 5430 microcentrifuge (Eppendorf AG, Hamburg, Germany), filtered through 0.45 µm PVDF filters, and the resulting extracts were analysed by rapid resolution liquid chromatography (RRLC) 1200 (Agilent Technologies, Mississauga, ON, Canada) coupled with a DAD-UV/Vis detector, using specific chromatographic conditions for each metabolite. The mobile phase was pumped at a flow rate of 1 mL/min. Quantification was performed using calibration curves with analytical standards of 0.1 mg/mL. Each sample was processed in triplicate and analysed in duplicate, with results expressed as mg/100 g dry weight.

Vitamin C was extracted using 1.2 mL of 3% metaphosphoric acid as a stabilising agent and 0.2 mL of 0.2% *DL*-homocysteine as a reducing agent, followed by homogenisation, sonication for one minute and adjustment of the volume to 2 mL. The extracts were filtered and analysed by RRLC with detection at 244 nm on a Zorbax Eclipse C18 column (1.8 µm, 4.6 mm × 50 mm) (Agilent Scientific Instruments, Santa Clara, CA, USA), using a mobile phase based on 1.5% monobasic potassium phosphate and 1.8% n-acetyl-n,n,n-trimethylammonium bromide, dissolved in methanol (90:10, *v*/*v*) and an injection volume of 20 µL. Quantification was performed using *L*-ascorbic acid as a standard.

The organic acid profile was determined by extraction with 1.5 mL of 0.02 N sulphuric acid in the presence of 0.05% metaphosphoric acid and 0.2% *DL*-homocysteine, followed by sonication for 3 min, adjustment to a volume of 2 mL and filtration. The extracts were analysed by RRLC with detection at 210 nm on a YMC-Triart C18 column (3 µm, 4.6 mm × 150 mm) (YMC Europe GmbH, Dinslaken, Germany), using a mobile phase based on 0.027% sulphuric acid and an injection volume of 20 µL. Quantification was performed using malic, citric, and tartaric acid standards injected separately.

Carotenoids were determined by extraction using organic solvents of varying polarity (250 µL of methanol, 500 µL of chloroform and 250 mL of water), followed by sonication for 2 min, recovery of the pigment fraction by centrifugation, and two successive re-extractions with 500 µL of chloroform. The extract was concentrated by evaporation at a temperature below 40 °C in a Büchi R-100 rotary evaporator (Fisher Scientific, Hampton, NH, USA). The dry extract was reconstituted with 40 µL of ethyl acetate and analysed by RRLC with detection between 250 and 500 nm on a YMC C30 column (3 µm, 4.6 × 150 mm) (YMC Europe GmbH, Dinslaken, Germany), using a mobile phase of acetonitrile (A), methanol (B) and ethyl acetate (C), with a linear gradient of 0–5 min, 85% A + 15% B; 5–7 min, 60% A + 20% B + 20% C; 7–12 min, 85% A + 15% B. The injection volume ranged from 3 to 20 µL. Quantification was performed using carotenoid standards such as astaxanthin, β-carotene, β-cryptoxanthin, lutein, lycopene, zeaxanthin, trans-β-apo-8′-carotenal, and α-carotene.

The analysis of phenolic compounds was carried out by extraction with 1 mL of 80% methanol acidified with 0.1% HCl, followed by sonication for 3 min, recovery of the liquid fraction by centrifugation, and two successive re-extractions with 500 µL of acidified methanol. The extract was filtered and analysed by RRLC with detection between 200 and 500 nm on a Zorbax Eclipse Plus C18 column (4.6 × 150 mm, 5 µm) (Agilent Scientific Instruments, Santa Clara, CA, USA), using a mobile phase of 0.01% formic acid (A) and acetonitrile (B) with a linear gradient of 0 min, 100% A; 5 min, 95% A + 5% B; 20 min, 50% A + 50% B; 30 min. The injection volume ranged from 3 to 20 µL. Quantification was performed using phenolic standards such as caffeic acid, chlorogenic acid, chrysin, *p*-, *m*- and *o*-coumaric acid, ferulic acid, gallic acid, *p*-hydroxybenzoic acid, 3-hydroxybenzoic acid, 2-methoxybenzoic acid, 3-methoxybenzoic acid, 2,5-dihydroxybenzoic acid, kaempferol, luteolin, naringin, quercetin, rutin, shikimic acid, syringic acid and vanillic acid.

### 2.3. Antioxidant Activity Analyses

Antioxidant activity was determined using 20 mg of a lyophilised sample extracted with 2 mL of methanol, sonicated for 3 min, centrifuged at 14,000 rpm for 4 min, and filtered through a 0.45 µm PDVF filter. Antioxidant capacity was assessed using the ABTS•^+^ and DPPH• free radical scavenging assays.

For the ABTS method, the radical was generated by reacting ABTS (7 mM) with potassium persulfate (2.45 mM), with the mixture kept in the dark at room temperature for 16 h. Subsequently, the solution was diluted with HPLC-grade methanol until an absorbance of 0.70 at 734 nm was reached. For the analysis, 10 µL of the extract or standard was mixed with 200 µL of the radical solution in a 96-well microplate, including reagent blanks. Absorbance was recorded at 734 nm using a microplate reader. Quantification was performed using a calibration curve with Trolox as the standard over the range 0.2–0.7 mM.

In the DPPH assay, the radical was prepared by dissolving 10 mg of DPPH in 50 mL of HPLC-grade methanol. Twenty microlitres of the extract or standard were added to 280 µL of the radical solution in a microplate, with the corresponding blanks included. The mixture was incubated in the dark for 30 min, and the reading was taken at 515 nm. Quantification was performed using a Trolox standard curve in the range of 0.4–4.0 mM. The results obtained using both methods were expressed as mmol Trolox equivalents per 100 g dry weight.

### 2.4. Antimicrobial Activity Analyses

#### 2.4.1. Antibacterial Activity

The antimicrobial activity was assessed using the broth microdilution method in accordance with the guidelines of the Clinical and Laboratory Standards Institute (CLSI). A stock solution was prepared by dissolving 400 mg of the lyophilised extract in 2 mL of sterile distilled water. Two-fold serial dilutions were performed in sterile 96-well microplates previously loaded with 100 µL of sterile Brain Heart Infusion (BHI) broth per well. Subsequently, 100 µL of the stock solution was added to the first column, and 100 µL was sequentially transferred to each subsequent column, ensuring thorough homogenization at each step. In the penultimate column, 100 µL was discarded to maintain a uniform final volume across all wells.

Inoculation was performed by preparing a bacterial suspension initially adjusted to a 0.5 McFarland standard, corresponding to approximately 1.5 × 10^8^ CFU/mL. This suspension was subsequently diluted according to CLSI recommendations to obtain a working inoculum suitable for microdilution assays. From this adjusted suspension, 20 µL was added to each well, yielding a final bacterial concentration of approximately 5 × 10^5^ CFU per well in a total reaction volume of 120 µL. The last row of the microplate was allocated for the experimental controls, consisting of a positive inhibition control (BHI supplemented with the inoculum and streptomycin), a growth control (BHI with inoculum only), and a negative sterility control (BHI alone).

Microplates were incubated for 24 h at 37 °C for *Escherichia coli* ATCC 8739, *Staphylococcus aureus* ATCC 6538P, *Pseudomonas aeruginosa* ATCC 9027 and *Streptococcus mutans* ATCC 25175. After incubation, cell viability was evaluated by adding 20 µL of 2,3,5-triphenyltetrazolium chloride (TTC) to each well, followed by reincubation at 37 °C for 1–2 h in the dark. TTC functions as a redox indicator: metabolically active cells reduce the reagent via dehydrogenase activity, producing insoluble red formazan. The presence or absence of this red colouration allowed visual determination of the minimum inhibitory concentration (MIC). All assays were performed in triplicate to ensure reproducibility.

#### 2.4.2. Antifungal Activity

The antifungal activity was evaluated using the broth microdilution method in accordance with the guidelines of the Clinical and Laboratory Standards Institute (CLSI). A stock solution was prepared by dissolving 400 mg of the lyophilised extract in 2 mL of sterile distilled water. Two-fold serial dilutions were performed in sterile 96-well microplates previously loaded with 100 µL of sterile Yeast Extract Peptone Dextrose (YPD) broth per well. Subsequently, 100 µL of the stock solution was added to the first column, followed by sequential transfers of 100 µL to each subsequent column, ensuring thorough homogenization at each step. In the penultimate column, 100 µL was discarded to maintain a uniform final volume across the plate.

Inoculation was performed by preparing a fungal suspension initially adjusted to 0.5 McFarland, corresponding to approximately 1.5 × 10^6^ CFU/mL. This suspension was subsequently diluted according to CLSI recommendations to obtain a working inoculum appropriate for broth microdilution assays. From this adjusted suspension, 20 µL was dispensed into each well, yielding a final fungal concentration of approximately 2.5 × 10^3^ CFU per well in a total reaction volume of 120 µL. The last row of the microplate was designated for the experimental controls, comprising a positive inhibition control (YPD supplemented with the inoculum and fluconazole), a growth control (YPD with inoculum only), and a negative sterility control (YPD alone).

Microplates were incubated at 37 °C for 24 h for *Candida albicans* ATCC 1031 and *Candida tropicalis* ATCC 13803. Following incubation, cell viability was assessed by adding 20 µL of 2,3,5-triphenyltetrazolium chloride (TTC) to each well, then reincubating at 37 °C for 1–2 h in the dark. TTC functions as a redox indicator: metabolically active cells reduce the reagent via dehydrogenase activity, producing insoluble red formazan. The presence or absence of this characteristic red colouration enabled visual determination of the minimum inhibitory concentration (MIC).

To confirm the colourimetric results, Sabouraud Dextrose Agar (SDA) plates were prepared in Petri dishes and marked with a reference grid. After the initial 24 h incubation, 4 µL from each well was spotted onto the corresponding grid sections of the labelled plates. These plates were incubated at 37 °C for an additional 24 h, and the presence or absence of fungal growth was recorded, completing a total incubation period of 48 h for the Candida strains evaluated. All assays were performed in triplicate to ensure reproducibility.

### 2.5. Statistical Analysis

Statistical processing and data visualisation were carried out using RStudio (version 4.4.1), Statgraphics Centurion XVII, and SigmaPlot 14.0, enabling the organisation to interpret and visualise the results. The variability of the physicochemical parameters was estimated by calculating the standard deviation for each fruit type. Differences in the degree of ripeness were assessed using analysis of variance (ANOVA), with a significance level of 0.5 for each fruit under study.

To examine the multivariate structure of the data, including clustering patterns among samples and variables that contribute most to total variability, a principal component analysis (PCA) was applied. Before this, the variables were standardised by centring on the mean and scaling to unit variance, to avoid biases arising from differences in units of measurement. Additionally, a heatmap with hierarchical clustering was generated to represent the physicochemical profiles and biological activities according to fruit type and ripeness stage. The data were first averaged by fruit maturity group, excluding variables with no variation. The data were first averaged by fruit maturity group, excluding variables with no variation. Subsequently, z-score normalisation was applied, and clustering was performed using Euclidean distance and the complete linkage method. The samples were differentiated by colour coding according to species and degree of ripeness.

## 3. Results and Discussion

### 3.1. Physico-Chemical Characteristics

Table 1 presents the physicochemical properties (weight, length, equatorial diameter, soluble solids, titratable acidity, moisture, and ash) and mineral content (iron, potassium, magnesium, and sodium) of the pepper varieties at five stages of ripeness.

The weight of the varieties studied ranged from 0.8 g (Malagueta chilli in M4) to 483.5 g (Yellow big peppers in M5). The weight increased progressively in most varieties as they ripened. This increase can be attributed primarily to genetic factors associated with each cultivar, which determine morphological characteristics such as size, metabolic capacity, cell division and expansion, as well as the accumulation of water, sugars and other structural and storage metabolites during fruit development [24,25]. In turn, the longitudinal diameter ranged from 20.3 mm (Yellow Big Peppers on M1) to 108.6 mm (Yellow Big Peppers on M5). Maturity did not show any pattern of change. The equatorial diameter ranged from 7.4 mm (Malagueta chilli M4) to 90.4 mm (Yellow big peppers M2). The ripening pattern showed an increase in Habanero chilli, Jalapeño chilli, and Orange medium peppers.

The pH of the variables under study ranged from 4.7 (Habanero chilli M5) to 6.0 (Orange medium peppers M1 and Yellow medium peppers M1). No consistent trend was observed with respect to maturity; in fact, Malagueta chilli and Yellow big peppers showed slight decreases. Furthermore, Jalapeño chilli increased up to M4, while other varieties fluctuated without a defined pattern. Thus, studies have indicated that the Jalapeño chilli has a slightly acidic pH, with reported ranges between 4.84 and 5.36, indicating that the pepper maintains a low acidity level [26], as shown in this study.

Titratable acidity ranged from 0.1% (Jalapeño chilli at M1 and M3, and Yellow medium peppers at M1) to 0.7% (Malagueta chilli at M3 and M4). There was no marked trend in acidity with degree of maturity; thus, Jalapeño chilli tended to increase toward M5, Orange Cherry peppers showed a slight decrease, Orange medium peppers ended with a marked increase at M5, and Yellow big peppers remained practically stable. Thus, these results were consistent with those of other authors who found that in a red *Capsicum* variety at different stages of phenological development, titratable acidity increased up to 50 days and then decreased. This behaviour suggests that the change in acidity during ripening does not follow a strictly linear trend, but rather responds to metabolic changes associated with the physiological development of the fruit. During the initial stages of growth and cell expansion, the accumulation of organic acids tends to increase due to high metabolic activity and their roles in energy metabolism and osmotic regulation. Subsequently, as ripening progresses, these acids may decrease as a result of their use as respiratory substrates or their conversion into other metabolic pathways, leading to progressive reductions in titratable acidity [27].

Soluble solids ranged from 2.7 °Brix (Yellow medium peppers at M1) to 14.0 °Brix (Habanero chilli at M2). The maturity trend was partially consistent in some sweet or semi-sweet varieties, but not in all. For example, Orange medium peppers increased toward M5, while Yellow medium peppers rose until M3, then declined; Orange Cherry peppers remained nearly stable; and Habanero chilli showed an early peak at M2, followed by a gradual decline. This indicates that solute accumulation does not follow a single kinetics, likely due to differences in respiration, sugar translocation, and metabolic use of carbohydrates, as suggested by other authors who note that during development, acids are synthesised from sugars and, as maturity progresses, these acids can be broken down through the Krebs cycle to release the energy necessary for fruit growth [28]. Other studies on *Capsicum* genotypes have indicated that total soluble sugar content in Capsicum fruits varies across ripening stages, tending to decrease as the fruit matures [1]. This behaviour demonstrates that the development of soluble solids during ripening does not follow a uniform pattern across different Capsicum genotypes, as indicated by a study of the ʹdedo-de-mocaʹ variety at different stages of phenological development, which showed that soluble solids increase up to 70 days and then decrease [27].

Moisture content ranged from 66.9% (Malagueta chilli M5) to 94.2% (Orange medium peppers in M3). The most pronounced trend was observed in Malagueta chilli, where moisture content decreased markedly as the fruit ripened. In Habanero chilli, there was also a moderate overall reduction, while in larger sweet varieties, moisture content remained more stable, especially in Yellow big peppers. Meanwhile, ash content peaked in Malagueta chilli M3 (4.4%) and reached its lowest level in Jalapeño chilli M2 (0.3%). Most varieties did not show a defined pattern of change. These results are consistent with those reported by other authors, who note that although each variety has a characteristic range upon reaching maturity, the final mineral composition depends closely on the genotype, soil type, mineral nutrition, and environmental conditions of the crop [26]. In turn, studies on bell peppers have documented that green (unripe) fruits have the highest moisture content (91.8%), which decreases significantly upon reaching the yellow (86.6%) and orange (86.3%) stages [29].

Iron showed the greatest relative range among minerals, with the highest value in Malagueta chilli in M1 (124.1 mg/100 g DW) and the lowest in Jalapeño chilli in M4 and M5 (0.1 mg/100 g DW). Potassium was the predominant mineral, with the lowest concentration in Malagueta chilli in M5 (3431.5 mg/100 g DW) and the highest in Orange Cherry peppers in M2 (12,316.0 mg/100 g DW). Magnesium reached a maximum in Habanero chilli in M5 (1239.0 mg/100 g DW) and a minimum in Orange cherry peppers in M3 (97.4 mg/100 g DW). Regarding the trend, Yellow medium peppers showed a final increase, while Yellow big peppers tended to decrease, and other varieties fluctuated with maturity. Sodium reached its highest value in Habanero chilli M2 (81.6 mg/100 g DW) and its lowest in Malagueta chilli M5 (6.2 mg/100 g DW). No uniform pattern of change was observed; thus, Orange medium peppers and Yellow medium peppers increased until advanced stages, while Habanero and Jalapeño fluctuated strongly. No clear pattern of change was observed in relation to fruit maturity. Thus, peppers are a rich source of essential trace and macrominerals, with potassium, iron, copper, and manganese standing out as key elements in the *Capsicum* genus [30].

### 3.2. Analysis of Bioactive Compounds

Table 2 presents of vitamin C, organic acids (citric, malic and tartaric acid), anthocyanins, and capsaicin, as well as the antioxidant activity measured by the ABTS and DPPH methods for pepper varieties.

Vitamin C levels ranged from 17.1 mg/100 g DW (Malagueta chilli M1) to 10,319.5 mg/100 g DW (Yellow medium peppers). In terms of behaviour during ripening, an increase in concentration was observed, primarily in Malagueta chilli. Similarly, Jalapeño chilli showed a general upward trend toward the M5 stage, although with intermediate fluctuations. In contrast, Orange cherry peppers showed a progressive decrease in vitamin C content throughout the stages evaluated. These results indicate the absence of a uniform pattern of vitamin C accumulation or degradation based on fruit maturity. Instead, the observed trends appear to be strongly influenced by genetic variability among cultivars. In this context, previous studies on peppers have reported that vitamin C concentration may increase during phenological development [31]; however, this behaviour depends on factors such as cultivar, harvest date, and growing conditions [32], as highlighted in a study conducted in Spain on the effect of fertilisation, irrigation and microbial biostimulants on the antioxidant profile of certain sweet pepper genotypes [31]. Other studies showed that unripe yellow and Orange Capsicum varieties exhibited high concentrations of vitamin C at the immature stages [28], as reported in a study on Orange cherry peppers, Yellow medium peppers and Yellow big peppers. This pattern is attributed to the antioxidant activity of vitamin C, which increases with rising respiratory rate during the peak phase of fruit development [1]. Other studies reported similar levels of vitamin C across the varieties studied [33].

Citric acid ranged from 68.6 mg/100 g DM (Malagueta chilli M3) to 2415.6 mg/100 g DW (Jalapeño chilli M4). Malic acid ranged from 234.9 mg/100 g DW (Malagueta chilli M3) to 4740.4 mg/100 g DW (Yellow medium peppers M4). Tartaric acid ranged from 14.5 mg/100 g DW (Jalapeño chilli M1) to 2673.4 mg/100 g DW (Jalapeño chilli M5). Overall, total organic acids ranged from 357.8 mg/100 g DW (Malagueta chilli M3) to 5298.1 mg/100 g DW (Yellow medium peppers M4). Regarding their behaviour during ripening, no single pattern of change was observed. In Yellow big peppers, total organic acids gradually decreased from M1 to M5. In Jalapeño chilli, they increased up to M4 and decreased slightly in M5. In Yellow medium peppers, the maximum was reached in M4. These results demonstrate that ripening affects the balance between the synthesis, accumulation, and degradation of organic acids, with a marked dependence on genotype. This variability can be explained by differences in respiratory rate and in the metabolic dynamics of the Krebs cycle, in which organic acids serve as key intermediates and can be utilised as substrates during the ripening process. Consistent with this, previous studies have indicated that the content of organic acids in peppers varies with the phenological stage; however, these changes do not necessarily follow a linear trajectory but depend on the cultivar and growing conditions [28,32].

Total anthocyanins ranged from 0.6 mg C-3-gl/100 g DW (Orange medium peppers M4) to 367.5 mg C-3-gl/100 g DW (Orange medium peppers M4), excluding non-detectable values. No clear trend in behaviour was observed during ripening. In Orange medium peppers, anthocyanin content increased progressively up to M4, then decreased markedly at M5. In contrast, in Yellow big peppers, a continuous reduction was observed as fruit maturity progressed. These results suggest that anthocyanin accumulation is primarily modulated by genetic and physiological factors rather than by a uniform pattern associated with ripeness. In this context, pigment regulation appears to depend on both fruit colour and developmental stage. Consistently, studies on coloured peppers have reported that the accumulation of pigments and antioxidant compounds is strongly influenced by genotype, fruit colour, and phenological stage, and does not necessarily follow a linear trend during ripening [34]. Whilst other studies on pepper genotypes indicate that the highest levels of anthocyanins were recorded in both mature and immature stages in red genotypes [1].

Capsaicin was detected only in Malagueta, Habanero, and Jalapeño. The highest concentration was found in Malagueta chilli M5 (1949.8 mg/100 g DW), while the lowest detectable concentration was in Habanero chilli M5 (165.5 mg/100 g DW). Regarding its behaviour during ripening, distinct patterns were observed among the varieties. In Malagueta chilli, the capsaicin concentration increased markedly up to M5. Similarly, Jalapeño chilli reached its maximum value at M5, although with intermediate fluctuations. In contrast, Habanero chilli showed a progressive decrease from the initial stages (M1–M2) toward M5. These results indicate that pungency, determined by the accumulation of capsaicinoids, does not follow a uniform pattern of increase with fruit ripening but rather depends on the genotype. The absence of capsaicin in sweet peppers is to be expected, given that the biosynthesis of these compounds is characteristic of hot varieties. According to the literature, capsaicinoids not only enable chemical differentiation between hot and non-hot materials within the genus Capsicum but also contribute to the antioxidant capacity of these fruits, thereby reinforcing their functional and nutritional value [35].

Antioxidant activity measured using the DPPH method ranged from 2.2 mmol ET/100 g DW (Malagueta chilli M3) to 41.3 mmol ET/100 g DW (Habanero chilli M2), while the ABTS method showed a range from 1.1 mmol ET/100 g DW (Orange medium peppers M1) to 6.8 mmol ET/100 g DW (Habanero chilli M2). The differences observed between the two methods can be attributed to the nature of the matrix and the reaction principles of each assay, as has been reported in the literature. In this regard, DPPH and ABTS exhibit different affinities for hydrophilic and lipophilic compounds, which influence the measured antioxidant response. These results reinforce the notion that antioxidant activity cannot be explained solely by the presence of vitamin C or capsaicin. Rather, there is likely a synergistic effect among various bioactive compounds, including phenols, carotenoids, anthocyanins, and other metabolites with reducing capacity, which collectively contribute to the fruit’s total antioxidant capacity [36]. Furthermore, other studies have indicated that yellow varieties exhibit greater antioxidant activity than red ones [1]; however, in this study, no significant changes were observed that would corroborate the previous results reported by other authors.

The main carotenoids identified in the bell pepper varieties were α-carotene, β-carotene, cantaxanthin, astaxanthin, capxanthin, β-cryptoxanthin, violaxanthin, luteoxanthin, zeaxanthin, lutein and zeinoxanthin. The total carotenoid content showed marked variability among genotypes and maturity stages. The highest value was recorded in Orange medium peppers at stage M5 (9495.8 mg/100 g DW), primarily due to high accumulation of capsanthin, β-carotene, and canthaxanthin. This behaviour is consistent with the physiology of the genus Capsicum, where, during ripening, the transition from chloroplasts to chromoplasts occurs, accompanied by the accumulation of carotenoids responsible for yellow, orange, and red colouration. The results for the Malaqueta chilli and Yellow big peppers are similar to those reported by other authors, who observed an accumulation of carotenoids with increasing ripeness in Capsicum genotypes [1]. Thus, in ripe pepper fruits, the predominant carotenoids include capsanthin, β-carotene, β-cryptoxanthin, zeaxanthin, violaxanthin, and lutein [37].

Table 3 presents the carotenoid profiles of the varieties studied for pepper varieties, at five stages of ripeness.

Regarding maturity, not all varieties followed a uniform pattern. Orange medium peppers showed the clearest trend, with total carotenoids increasing from 98.0 mg/100 g DW at M1 to 9495.8 mg/100 g DW at M5. A similar pattern was observed in Malagueta chilli and Jalapeño chilli, where carotenoids increased markedly toward M5. In contrast, Yellow big peppers showed a progressive decrease from M1 to M5, indicating that ripening does not always imply a net accumulation of carotenoids. These differences can be attributed to factors such as genotype, final fruit colour, differential expression of genes involved in carotenoid biosynthesis, oxidative degradation, xanthophyll esterification, and physiological status at harvest [38].

β-carotene was found in particularly high concentrations in Orange medium peppers (M5) and Yellow medium peppers (M3). This compound is nutritionally significant because it acts as a provitamin A carotenoid. Similarly, β-cryptoxanthin also exhibits provitamin A activity, although its concentrations were considerably lower. From a functional perspective, these varieties could constitute important sources of carotenoids with potential benefits for visual health, the immune system, and antioxidant capacity [37].

Table 4 presents the phenolic profiles (phenolic acids and individual flavonoids) of the pepper varieties studied, at five stages of ripeness.

The main phenolic compounds identified in the sweet and hot pepper varieties were gallic acid, 4-hydroxybenzoic acid, catechin, vanillic acid, *m*-coumaric acid, syringic acid, chlorogenic acid, caffeic acid, naringenin, ferulic acid, sinapic acid, kaempferol, quercetin glucoside, and quercetin. The results of this study are consistent with those reported by other authors, who note that chilli peppers contain polyphenols and capsaicinoids with antioxidant, anti-inflammatory, and metabolic activities as described in in vitro and in vivo models [39].

No universal trend of increasing or decreasing phenolic content with maturity was identified. Malagueta chilli increased up to M4 and decreased at M5, suggesting maximum accumulation at an advanced but not terminal stage. Habanero chilli showed a progressive decrease in total phenols from M1 to M5. Orange Cherry peppers peaked at M3; Orange medium peppers peaked at M2 and then decreased; Yellow big peppers increased up to M3 and subsequently decreased. This behaviour is consistent with reports on the genus Capsicum, where phenolic content depends on genotype, ripeness stage, cultivation system, and environmental conditions [40]. Other studies indicate an increase in phenolic compounds depending on the degree of ripeness in hot pepper varieties [41].

In green and red sweet peppers, it has been reported that the stage of ripeness significantly affects the phenolic profile and antioxidant activity. Still, the direction of the change depends on the compound and the cultivar [41]. In Yellow big peppers, the increase up to M3 and subsequent decline suggest that intermediate stages might be more favourable for phenolic accumulation. This pattern partially aligns with studies reporting higher concentrations of certain phenols in immature or intermediate stages than in fully ripe stages [40].

### 3.3. Antimicrobial Activity Analyses

Table 5 presents the antimicrobial activity of dried ethanolic extracts from seven varieties of *Capsicum* at five stages of ripeness, as determined by the minimum inhibitory concentration against bacteria (*Escherichia coli*, *Staphylococcus aureus*, *Pseudomonas aeruginosa*, *Streptococcus mutans*) and fungi (*Candida albicans*, *Candida tropicalis*).

Antimicrobial activity against *Escherichia coli* varied among genotypes and stages of ripeness, with no consistent trend observed. The highest activity was observed in Yellow medium peppers at M4, with an MIC of 2.6 mg/mL. In comparison, the highest active value (lowest activity) was 41.7 mg/mL, recorded in Orange cherry peppers (M3), Yellow medium peppers (M2), and Yellow big peppers (M2–M3). These results are consistent with previous reports indicating lower susceptibility in Gram-negative bacteria; however, capsaicin isolated from Malagueta has been reported to exhibit MIC values of 5 µg/mL against *E. coli* [42,43]. This reduced susceptibility may be due to the outer membrane of Gram-negative bacteria being rich in lipopolysaccharides, which act as a selective barrier that limits the entry of bioactive compounds present in plant extracts. Therefore, the variable activity observed between genotypes and stages of ripeness probably does not depend on a single metabolite, but rather on the distinct composition of antimicrobial compounds accumulated at each stage of ripeness, as suggested by other authors [44].

In *Staphylococcus aureus*, the highest activity was observed with orange medium peppers in M4 (5.2 mg/mL), while the highest inhibitory concentration was 70.2 mg/mL for Habanero chilli in M5. The higher relative sensitivity of this Gram-positive bacterium is consistent with the literature, which reports greater susceptibility to phenolic compounds and capsaicinoids due to the absence of a complex outer membrane. Furthermore, it has been reported that capsaicin isolated from Malagueta may exhibit an MIC of 1.2 µg/mL against *S. aureus* [42,43].

In the case of *Pseudomonas aeruginosa*, the best activity was observed in Orange cherry peppers in M5 (20.8 mg/mL), while the highest active concentration was 65.1 mg/mL in Jalapeño chilli in M3. Limited activity against this microorganism is to be expected, given that it possesses a poorly permeable outer membrane and intrinsic resistance mechanisms that restrict the action of plant extracts. However, an MIC of 10 µg/mL has been reported for isolated capsaicin against *P. aeruginosa* [42,45].

Furthermore, in *Streptococcus mutans*, the highest activity was observed with Jalapeño chilli at 2.0 mg/mL in stages M1, M2, M3, and M5. Orange medium peppers also stood out in M2 (2.6 mg/mL). The highest activity value was observed for Malagueta chilli in M4 (62.9 mg/mL), again demonstrating a response dependent on genotype and stage of maturity.

As for fungi, *Candida albicans* showed the highest susceptibility in Yellow medium peppers at M1 (10.5 mg/mL), while the highest active concentrations were 41.7 mg/mL in Orange cherry peppers (M3), Orange medium peppers (M2), and Yellow big peppers (M2). For *Candida tropicalis*, the best activity was observed in Orange medium peppers at M1 (20.9 mg/mL), with maximum values of 41.7 mg/mL in Orange cherry peppers (M3) and Yellow big peppers (M2). It has consistently been reported that isolated capsaicin can exhibit an MIC of 25 µg/mL against *C. albicans* [42].

These results are consistent with reviews of the genus *Capsicum*, in which extracts obtained from different tissues and solvents have demonstrated activity against bacteria and fungi, including *Staphylococcus aureus*, *Streptococcus pyogenes*, *Salmonella typhi*, *Bacillus cereus*, and *Aspergillus flavus*. However, the magnitude of the antimicrobial response depends on the microorganism, the genotype, the solvent used, and the extract concentration, which explains the variability observed in this study [39,46].

*Capsicum* fruits have been reported to contain a wide range of bioactive compounds, including carotenoids, capsaicinoids, phenolic compounds, flavonoids, organic acids, and vitamin C. Among the compounds evaluated in the present study, capsaicinoids, particularly capsaicin, organic acids, and phenolic compounds may contribute to the antimicrobial activity observed against the tested microorganisms. *Capsaicin* has been associated with alterations in the microbial cell envelope, including disruption of membrane integrity, increased permeability, leakage of intracellular components, and interference with essential cellular processes [47]. These effects may ultimately compromise cell viability.

Organic acids may also contribute to microbial inhibition through multiple mechanisms. In their undissociated form, these compounds can diffuse across the microbial cell membrane and dissociate within the cytoplasm, leading to intracellular acidification and disruption of pH homeostasis. This process can interfere with the proton motive force, membrane transport, motility, and ATP synthesis, thereby affecting essential metabolic functions. In addition, acidic intracellular conditions may inhibit key enzymes involved in microbial metabolism and induce damage to cellular macromolecules, including DNA, ultimately contributing to growth inhibition or cell death [48,49].

Phenolic compounds may further enhance antimicrobial effects through their interaction with microbial membranes and intracellular targets. These compounds have been related to reversible perturbations in membrane permeability, disruption of membrane structure, leakage of cellular contents, and interference with enzyme activity and oxidative balance [44].

In the case of fungi/yeasts, higher MIC values were observed, suggesting lower susceptibility to the tested extracts compared with bacterial strains. This response may be associated with the intrinsic structural and physiological complexity of fungal cells. Fungi possess a eukaryotic organisation and a rigid cell wall, primarily composed of β-glucans, chitin, and glycoproteins, which may act as a protective barrier, reducing the effective interaction of bioactive compounds with intracellular targets [44]. In addition, the overexpression of efflux pumps, particularly ATP-binding cassette (ABC) and major facilitator superfamily (MFS) transporters, has been described as an important mechanism of antifungal resistance, as these systems can actively export xenobiotics and antifungal compounds, thereby reducing their intracellular accumulation [50].

Furthermore, fungi exhibit considerable metabolic versatility, in part due to their ability to produce extracellular enzymes that degrade or transform complex organic substrates. This enzymatic repertoire may contribute to their adaptation to chemically complex environments, such as those generated by plant extracts containing compounds with different polarity, molecular size, and biological targets [51]. In comparative ecological contexts, bacteria are often more efficient in the uptake and utilisation of simple substrates, whereas fungi tend to show greater relative capacity to exploit complex organic matter [52]. This metabolic flexibility, together with cell wall-mediated protection and active efflux mechanisms, may help explain the higher MIC values observed against fungal strains.

### 3.4. Statistical Analysis

**Figure 3:**
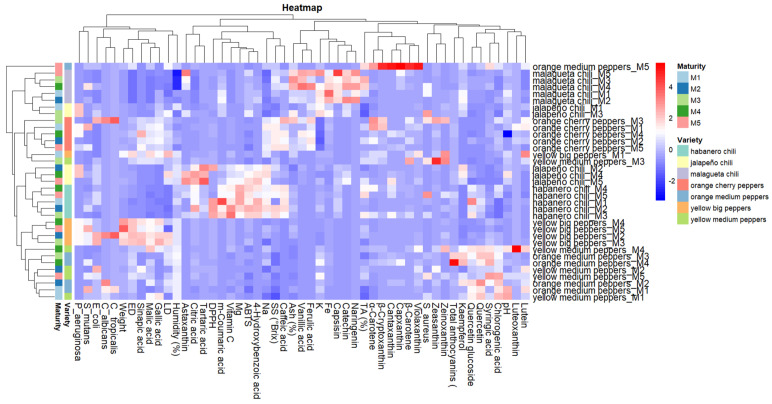
Heatmap of the variables under study, considering the varieties and stages of ripeness under study. Note: LD, longitudinal diameter; ED, equatorial diameter; Mg, magnesium; ABTS, antioxidant activity by ABTS; DPPH, antioxidant activity by DPPH; Na, sodium; SS, soluble solids; K, potassium; Fe, iron; TA, total titratable acidity. M1, maturity at 0%; M2, maturity at 10%; M3, maturity at 50%; M4, maturity at 80%; M5, maturity at 100%.

Figure 3 shows the hierarchical heatmap of the physicochemical variables, minerals, bioactive compounds, antioxidant activity, and antimicrobial activity of the six pepper varieties under study at five stages of ripeness. In this representation, rows correspond to variety–ripeness combinations, while columns include the different analytical variables.

The clustering pattern reveals a clear separation among varieties, suggesting that genotype exerts a stronger influence than ripening stage on the overall biochemical profile. Specifically, Malagueta chilli is primarily associated with elevated values of capsaicin, certain carotenoids, and selected phenolic compounds. In contrast, Habanero chilli is characterised by high levels of vitamin C, antioxidant activity, and specific minerals. Meanwhile, Orange medium peppers and Yellow medium peppers form clusters linked to carotenoids, anthocyanins, and flavonoids. This distribution is consistent with previous studies indicating that metabolite composition in *Capsicum* is strongly influenced by genotype, ripening stage, light exposure, harvest season, and cultivation conditions [53].

Within the bioactive compound block, a close association is observed among capsaicin, total carotenoids, capsanthin, β-carotene, zeaxanthin, and antioxidant activity, particularly in mature samples of pungent or orange-coloured varieties. This relationship is biologically coherent, as ripening in *Capsicum* involves the transition from chloroplasts to chromoplasts, accompanied by the accumulation of coloured carotenoids. Additionally, studies have reported positive correlations between capsaicinoids, phenolic compounds, and antioxidant activity, especially when evaluated using ABTS and FRAP assays [54].

The cluster corresponding to phenolic compounds and flavonoids shows proximity among chlorogenic acid, caffeic acid, ferulic acid, quercetin, quercetin glycoside, kaempferol, and naringenin. This grouping aligns with the phenylpropanoid pathway, as these compounds share common metabolic precursors. Their distribution does not follow a linear trend with ripening but instead exhibits variety-specific peaks associated with physiological stages. Previous studies confirm that phenolic acids and flavonoids in *Capsicum* are modulated by ripening, radiation, and harvest season, with responses strongly dependent on genotype [53]. Furthermore, *Capsicum* has been described as a rich matrix of vitamin C, carotenoids, phenolics, and capsaicinoids, whose profiles can vary substantially across cultivars and environmental conditions. Studies involving multiple cultivars have shown that antioxidant activity is positively associated with capsaicinoids and that phenolic composition significantly contributes to overall bioactivity, supporting the clustering observed between pungent compounds, phenolics, and antioxidant capacity [54].

Finally, the heatmap suggests that advanced ripening stages, particularly M4 and M5, tend to exhibit higher relative levels of carotenoids and certain pigments in specific varieties, notably *Orange medium peppers* and *Malagueta chilli*. In contrast, compounds such as lutein and some phenolics appear to be more concentrated in early or intermediate stages. This pattern is consistent with the expected metabolic shift during ripening, where chromoplastic carotenoids progressively replace photosynthetic pigments. Indeed, recent studies report that lutein predominates in immature fruits, whereas β-carotene increases during later stages of ripening [53].

**Figure 4:**
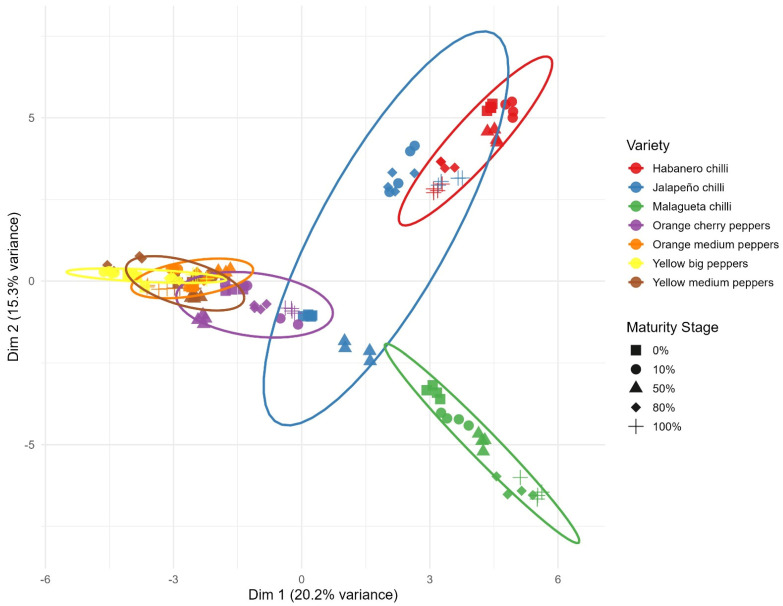
Principal component analysis of pepper varieties by degree of ripeness. Note: M1, maturity at 0%; M2, maturity at 10%; M3, maturity at 50%; M4, maturity at 80%; M5, maturity at 100%.

Figure 4 shows the principal component analysis (PCA) of bell pepper varieties based on maturity. Dimension 1 (Dim 1) explains 20.2% of the variance, and Dimension 2 (Dim 2) explains 15.3%, accounting for 35.5% of the total variability. Therefore, the PCA accounts for only a moderate fraction of the variation in the dataset.

The distribution pattern shows a clearer separation by variety than by ripeness stage. Habanero chilli clusters are in the upper right quadrant, while Malagueta chilli is in the lower right quadrant. Meanwhile, Yellow big peppers and Yellow medium peppers are concentrated toward negative values of Dim1, indicating relatively similar compositional profiles. Orange Cherry peppers occupy an intermediate position with a negative trend, while Jalapeño chilli exhibits the widest dispersion, suggesting greater internal heterogeneity among its ripeness stages.

The distinction between Habanero chilli and Malagueta chilli indicates that, despite sharing pungent characteristics, their physicochemical and bioactive profiles differ significantly. In contrast, the similarity between Yellow big peppers and Yellow medium peppers suggests more homogeneous compositions, possibly associated with lower capsaicinoid content and characteristics typical of sweet or semi-sweet peppers.

These results are consistent with previous studies on the genus *Capsicum*, in which multivariate analyses have shown that genotype accounts for a significant proportion of phytochemical variability, alongside factors such as ripeness, genotype, environment, storage conditions and processing [1]. In particular, it has been reported that cultivars can be distinguished by compounds such as capsaicinoids, phenols, and other bioactive metabolites, thereby supporting the structuring role of genetic variability in the chemical profile [55]. Furthermore, it has been reported that *Capsicum* fruits contain a wide range of bioactive compounds, including carotenoids, capsaicinoids, phenols, flavonoids, and vitamin C, whose variability contributes to the observed differentiation among varieties when multivariate analysis tools are applied [39].

**Figure 5:**
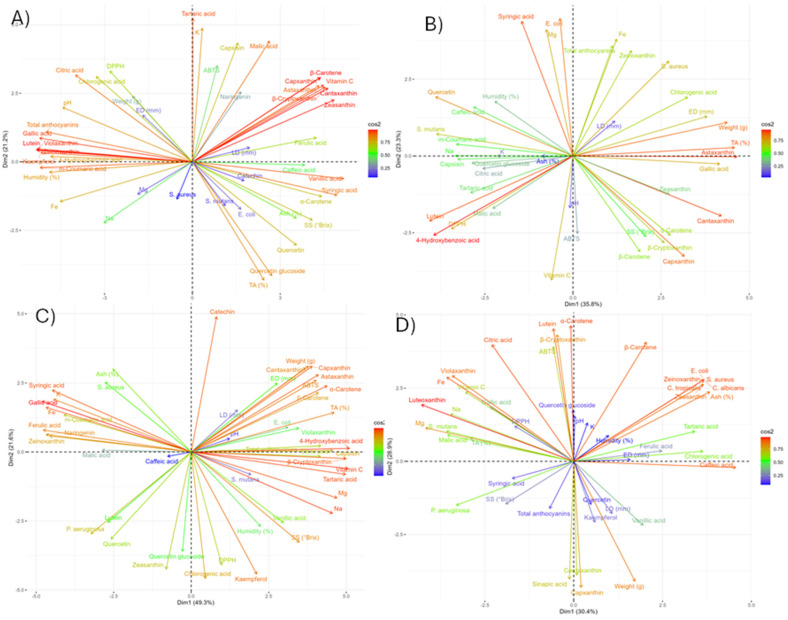

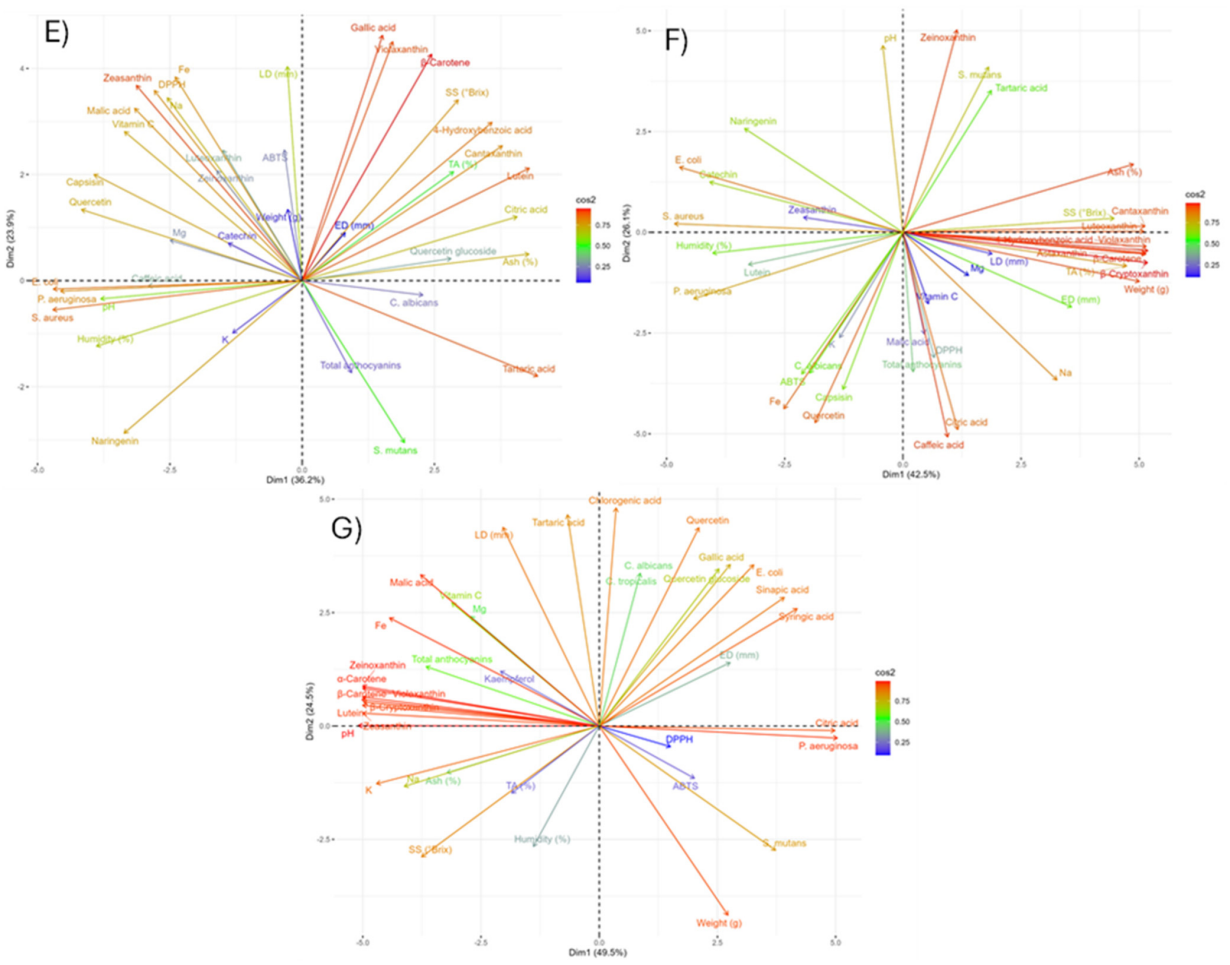
Principal component analysis by variety under study. Note: (**A**), Malagueta chilli (*Capsicum frutenses* L.); (**B**), Habanero chilli (*Capsicum chinense* Jacq.); (**C**), Jalapeño chilli (*Capsicum annuum* L.); (**D**), Orange cherry peppers; (**E**), Yellow medium peppers; (**F**), Orange medium peppers; (**G**), Yellow big peppers. LD, longitudinal diameter; ED, equatorial diameter; Mg, magnesium; ABTS, antioxidant activity by ABTS; DPPH, antioxidant activity by DPPH; Na, sodium; SS, soluble solids; K, potassium; Fe, iron; TA, total titratable acidity.

Figure 5 shows several principal component analyses (PCAs) constructed independently for each variety: (A) Malagueta chilli; (B) Habanero chilli; (C) Jalapeño chilli; (D) Orange cherry peppers; (E) Yellow medium peppers; (F) Orange medium peppers; and (G) Yellow large peppers. Each graph shows the relative contributions of physicochemical variables, minerals, bioactive compounds, antioxidant activity, and antimicrobial activity to the first two PCA dimensions.

In Malagueta chilli (Figure 5A), carotenoids, vitamin C, capsaicin, and ABTS activity cluster in the same quadrant, indicating a positive correlation between pigments, pungency, and antioxidant capacity. In contrast, moisture content and some antimicrobial variables cluster in the opposite quadrant, suggesting that matrices with higher water content have a lower relative concentration of bioactive compounds. This pattern is consistent with reports linking capsaicinoids and phenols to antioxidant activity in *Capsicum* [54].

In Habanero chilli (Figure 5B), vitamin C, capsaicin, β-carotene, and β-cryptoxanthin cluster along the same axis, while moisture, quercetin, and certain antimicrobial variables cluster in the opposite direction.

In Jalapeño chilli (Figure 5C), weight, ED, capsaicin, β-carotene, capsanthin, and organic acids cluster in the same quadrant, suggesting that morphological development may be associated with the accumulation of compounds involved in ripening and pungency. In contrast, quercetin and naringenin, along with their activity against *P. aeruginosa*, appear in the opposite quadrant, indicating distinct profiles between antioxidant and antimicrobial responses. This separation is consistent with reports showing that the antimicrobial activity of *Capsicum* does not depend on a single metabolite, but rather on complex mixtures of capsaicinoids, phenols, and flavonoids [56].

In Orange cherry peppers (Figure 5D), organic acids, vitamin C, carotenoids, and antifungal activity are found in distinct areas. In Yellow medium peppers (Figure 5E), the levels of phenols, gallic acid, naringenin, and soluble solids tend to cluster together. At the same time, total anthocyanins, minerals, and some antimicrobial variables show a more dispersed pattern. This suggests a profile that depends on maturity and distinct metabolic pathways. The literature confirms that *Capsicum* fruits contain capsaicinoids, carotenoids, phenols, and flavonoids, but their accumulation varies with genotype, developmental stage, and environment [57].

In Orange medium peppers (Figure 5F), weight, equatorial diameter, capsanthin, β-carotene, β-cryptoxanthin, canthaxanthin, and soluble solids cluster together. In Yellow big peppers (Figure 5G), carotenoids, vitamin C, Mg, and certain organic acids cluster in a different sector from weight and certain antimicrobial variables. In general, an inverse relationship is observed between moisture content and metabolite concentration, with more mature fruits having higher levels of soluble solids and bioactive compounds. This pattern has been reported in *Capsicum*, where increased metabolic activity during ripening is associated with a relative decrease in water content [58].

The antimicrobial and antioxidant activity of *Capsicum* extracts highlights their potential relevance for the food and health sectors. In food preservation, extracts containing phenolic compounds and organic acids could be considered as natural preservatives due to their ability to inhibit microbial growth, delay oxidative deterioration, and potentially extend product shelf life, reducing the use of synthetic additives [44,59]. In the health sector, these extracts may be of interest as alternative or adjunctive antimicrobial agents, particularly against microorganisms of clinical relevance, including multidrug-resistant strains [60].

Nevertheless, further studies evaluating bactericidal/fungicidal activity, cytotoxicity, selectivity, stability, and performance in real food or biological systems are necessary before proposing their practical application.

**Figure 6:**
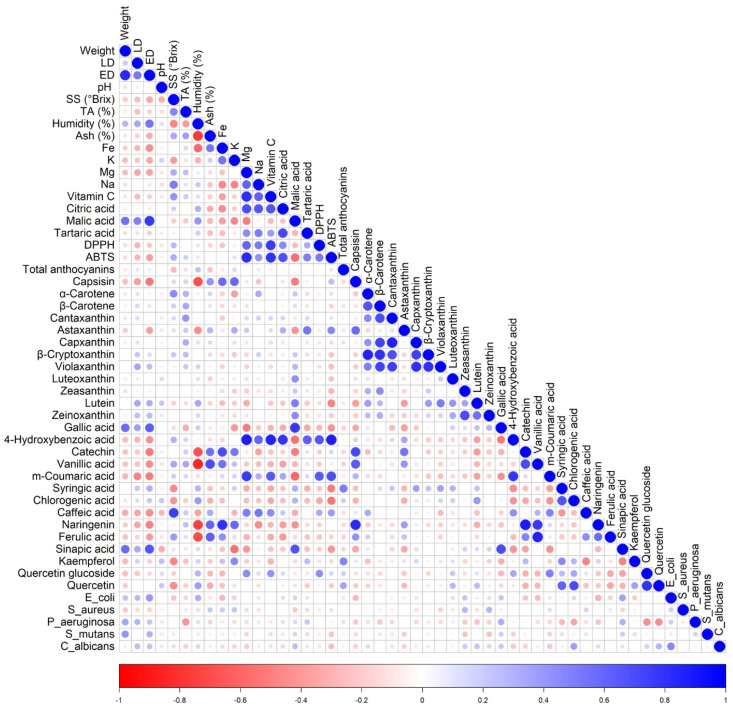
Pearson correlation matrix. Note: LD, longitudinal diameter; ED, equatorial diameter; SS, soluble solids; TA, Total titratable acidity; Fe, iron; K, potassium; Mg, magnesium; Na, sodium; ABTS, antioxidant activity by ABTS; DPPH, antioxidant activity by DPPH.

Figure 6 shows the Pearson correlation matrix between the physicochemical variables, mineral content, bioactive compounds, antioxidant activity and antimicrobial activity. The results indicated a positive correlation between weight and size, moisture content and size, Mg and vitamin C, Mg and citric acid, size and malic acid, Mg and ABTS, vitamin C and ABTS, citric acid and ABTS, and 4-hydroxybenzoic acid with Mg, vitamin C, citric acid, DPPH and ABTS. Furthermore, a positive correlation was observed between carotenoids and between phenolic compounds; this is very typical of metabolites from the same metabolic pathway [61].

The novelty of this work lies not only in the broad number of variables evaluated but also in the integration of nutritional, phytochemical, antioxidant, and antimicrobial datasets through multivariate approaches. Unlike previous studies that focused on isolated compounds or individual pepper types, the present research demonstrates that genotype exerts a stronger influence than ripening stage on the overall bioactive profile and biological activity of Capsicum fruits. This integrated approach provides a more complete understanding of the functional potential of sweet and hot peppers.

## 4. Conclusions

This study provides one of the most comprehensive assessments of sweet and hot peppers reported to date, integrating physicochemical parameters, mineral composition, multiple classes of bioactive compounds, antioxidant activity, antimicrobial activity, and multivariate analyses across five ripening stages. Although ripening modulated metabolite levels, no universal pattern of simultaneous increases or decreases was observed among the evaluated compounds. Among minerals, potassium was one of the most abundant, particularly in Malagueta chilli (M5). Regarding bioactive compounds, distinct genotype-dependent maxima were identified: Habanero chilli exhibited high vitamin C (M3); Yellow medium peppers showed elevated malic acid (M4); Orange medium peppers reached maximum total anthocyanins (M4) and carotenoids (β-carotene, canthaxanthin, capsanthin) in M5; Malagueta chilli presented the highest capsaicin content in M5; Yellow big peppers showed maximum gallic acid (M3); Habanero chilli had elevated *m*-coumaric acid (M1); Orange cherry peppers showed higher caffeic acid (M3); and Malagueta chilli exhibited increased naringenin (M2). Antioxidant activity across ripening stages was generally higher in hot varieties. Minimum inhibitory concentrations (MIC) were observed in Yellow medium peppers (M4) against *Escherichia coli*; Orange medium peppers (M4) against *Staphylococcus aureus*; and Jalapeño chilli (M1, M2, M3, M5) against *Streptococcus mutans*. Multivariate analyses (PCA and heatmap) confirmed that chemical and biological variability is primarily structured by genotype, revealing clusters associated with carotenoids, phenolics, and pungent compounds. The findings demonstrate that genotype is a stronger determinant of bioactive composition than fruit maturity, providing new evidence for selecting *Capsicum* varieties with enhanced functional and nutraceutical value. Overall, these findings lay the groundwork for future research aimed at optimising strategies for utilising genotypes with specific chemical characteristics, as well as evaluating processes for the extraction, purification and biological validation of metabolites with potential applications in the food, pharmaceutical and nutraceutical sectors.

## Figures and Tables

**Table 1:** Physicochemical and mineral content of the pepper varieties under study.

Common Name	Maturity	Weight (g)	LD (mm)	ED (mm)	pH	SS (°Brix)	TA (%)	Humidity (%)	Ash (%)	Fe (mg/100 g DW)	K (mg/100 g DW)	Mg (mg/100 g DW)	Na (mg/100 g DW)
Malagueta chili	M1	0.9 ± 0.1 ^a^	23.9 ± 0.1 ^d^	8.5 ± 0.1 ^a^	5.3 ± 0.0 ^a^	8.3 ± 0.5 ^b^	0.4 ± 0.1 ^b^	83.6 ± 0.7 ^a^	1.8 ± 0.4 ^c^	124.1 ± 7.2 ^a^	2701.8 ± 11.9 ^b^	177.0 ± 2.2 ^a^	21.1 ± 2.2 ^a^
	M2	0.9 ± 0.0 ^a^	36.3 ± 0.1 ^a^	8.5 ± 0.1 ^a^	5.2 ± 0.0 ^a^	6.3 ± 0.5 ^c^	0.3 ± 0.0 ^b^	81.2 ± 1.2 ^b^	2.0 ± 0.5 ^c^	88.6 ± 9.7 ^b^	2609.2 ± 32.7 ^c^	167.5 ± 34.6 ^a^	16.4 ± 2.7 ^b^
	M3	0.9 ± 0.0 ^a^	34.6 ± 0.2 ^b^	8.0 ± 0.1 ^b^	5.0 ± 0.0 ^b^	11.0 ± 0.0 ^a^	0.7 ± 0.1 ^a^	68.8 ± 3.5 ^c^	4.4 ± 1.1 ^a^	65.6 ± 7.5 ^d^	1819.8 ± 1.9 ^e^	157.2 ± 31.0 ^ab^	9.5 ± 0.6 ^c^
	M4	0.8 ± 0.1 ^a^	31.0 ± 0.1 ^c^	7.4 ± 0.1 ^c^	5.0 ± 0.0 ^b^	10.0 ± 0.0 ^a^	0.7 ± 0.1 ^a^	70.0 ± 5.0 ^c^	3.9 ± 0.7 ^ab^	75.4 ± 0.5 ^c^	2560.6 ± 13.6 ^d^	169.5 ± 17.5 ^a^	19.8 ± 1.9 ^ab^
	M5	0.9 ± 0.1 ^a^	34.1 ± 0.4 ^b^	8.4 ± 0.0 ^a^	5.1 ± 0.0 ^b^	9.3 ± 0.5 ^b^	0.3 ± 0.1 ^b^	66.9 ± 4.5 ^c^	3.1 ± 1.8 ^b^	47.6 ± 9.7 ^e^	3431.5 ± 29.2 ^a^	148.2 ± 0.7 ^b^	6.2 ± 1.3 ^c^
Habanero chili	M1	1.8 ± 0.5 ^c^	29.1 ± 3.3 ^a^	15.5 ± 1.6 ^b^	4.9 ± 0.0 ^a^	6.0 ± 0.7 ^c^	0.3 ± 0.0 ^b^	90.2 ± 0.6 ^a^	1.2 ± 0.2 ^b^	6.9 ± 0.3 ^b^	1889.1 ± 34.0 ^a^	1220.2 ± 17.5 ^a^	72.8 ± 8.1 ^b^
	M2	2.8 ± 0.2 ^b^	24.4 ± 5.5 ^b^	17.7 ± 3.0 ^b^	4.8 ± 0.0 ^ab^	14.0 ± 0.0 ^a^	0.3 ± 0.0 ^b^	85.8 ± 1.9 ^b^	1.5 ± 0.2 ^a^	7.1 ± 0.2 ^b^	2057.3 ± 38.1 ^a^	1002.7 ± 2.2 ^c^	81.6 ± 6.8 ^a^
	M3	3.9 ± 0.7 ^b^	27.1 ± 4.7 ^ab^	19.4 ± 2.3 ^ab^	4.9 ± 0.0 ^a^	13.5 ± 1.7 ^a^	0.6 ± 0.1 ^a^	85.2 ± 0.7 ^b^	1.3 ± 0.1 ^ab^	5.8 ± 0.2 ^c^	1824.1 ± 14.1 ^b^	730.2 ± 49.5 ^d^	61.5 ± 6.0 ^b^
	M4	5.0 ± 0.2 ^a^	29.7 ± 1.8 ^a^	22.2 ± 1.5 ^b^	4.8 ± 0.0 ^ab^	12.3 ± 0.5 ^b^	0.5 ± 0.1 ^a^	87.7 ± 0.3 ^a^	1.2 ± 0.1 ^b^	5.3 ± 0.0 ^c^	1830.0 ± 16.8 ^b^	1133.7 ± 32.7 ^b^	58.3 ± 8.0 ^b^
	M5	5.7 ± 0.3 ^a^	29.8 ± 2.4 ^a^	23.6 ± 1.4 ^a^	4.7 ± 0.0 ^b^	11.3 ± 0.5 ^b^	0.6 ± 0.0 ^a^	86.4 ± 0.2 ^ab^	1.4 ± 0.0 ^a^	12.2 ± 1.6 ^a^	1895.3 ± 88.0 ^a^	1239.0 ± 23.0 ^a^	63.1 ± 6.3 ^b^
Jalapeño chili	M1	9.7 ± 1.5 ^d^	43.3 ± 7.8 ^c^	22.3 ± 1.5 ^b^	5.0 ± 0.0 ^c^	5.0 ± 0.0 ^e^	0.1 ± 0.0 ^c^	92.0 ± 0.2 ^a^	0.8 ± 0.0 ^b^	32.5 ± 0.2 ^b^	2528.5 ± 18.0 ^b^	136.6 ± 0.5 ^e^	21.0 ± 2.5 ^c^
	M2	12.2 ± 0.6 ^c^	52.0 ± 4.1 ^c^	23.3 ± 2.4 ^b^	5.0 ± 0.0 ^c^	9.8 ± 0.5 ^a^	0.2 ± 0.0 ^b^	92.1 ± 0.1 ^a^	0.3 ± 0.0 ^c^	6.5 ± 0.1 ^c^	1921.4 ± 31.3 ^c^	712.5 ± 33.7 ^a^	77.5 ± 6.6 ^a^
	M3	16.6 ± 2.4 ^b^	77.0 ± 25.9 ^ab^	25.1 ± 2.7 ^ab^	5.3 ± 0.0 ^b^	6.0 ± 0.6 ^d^	0.1 ± 0.0 ^c^	79.6 ± 5.6 ^c^	2.8 ± 0.8 ^a^	42.6 ± 0.6 ^a^	2606.9 ± 29.1 ^a^	212.1 ± 15.5 ^d^	22.9 ± 0.1 ^c^
	M4	20.0 ± 1.2 ^a^	94.7 ± 32.3 ^a^	26.2 ± 3.1 ^a^	5.6 ± 0.0 ^a^	8.0 ± 0.0 ^b^	0.3 ± 0.0 ^ab^	91.5 ± 0.3 ^b^	0.8 ± 0.1 ^b^	0.1 ± 0.0 ^d^	1648.4 ± 29.5 ^d^	534.9 ± 29.3 ^c^	79.6 ± 11.3 ^a^
	M5	24.3 ± 2.4 ^a^	68.8 ± 1.3 ^b^	27.8 ± 1.5 ^a^	5.2 ± 0.0 ^b^	7.0 ± 0.0 ^c^	0.4 ± 0.0 ^a^	90.6 ± 0.3 ^b^	0.8 ± 0.1 ^b^	0.1 ± 0.0 ^d^	1935.8 ± 51.1 ^c^	660.1 ± 5.4 ^b^	65.1 ± 0.0 ^b^
Orange cherry peppers	M1	14.9 ± 3.0 ^c^	52.8 ± 6.8 ^b^	31.3 ± 2.5 ^b^	5.0 ± 0.0 ^ab^	12.8 ± 1.0 ^a^	0.4 ± 0.0 ^a^	82.3 ± 2.6 ^ab^	1.2 ± 0.3 ^c^	21.3 ± 1.8 ^a^	1354.9 ± 0.5 ^a^	125.7 ± 0.9 ^a^	74.3 ± 2.3 ^a^
	M2	19.5 ± 1.8 ^b^	62.9 ± 6.2 ^a^	33.5 ± 2.7 ^ab^	4.9 ± 0.0 ^b^	12.5 ± 1.4 ^a^	0.4 ± 0.1 ^a^	85.6 ± 1.1 ^a^	1.5 ± 0.3 ^bc^	14.8 ± 0.0 ^b^	1216.0 ± 27.8 ^b^	102.4 ± 2.1 ^b^	54.6 ± 3.7 ^c^
	M3	21.5 ± 2.1 ^b^	57.8 ± 3.7 ^ab^	37.2 ± 2.5 ^a^	5.1 ± 0.0 ^a^	11.6 ± 0.4 ^a^	0.3 ± 0.0 ^a^	84.0 ± 1.2 ^a^	3.5 ± 0.2 ^a^	14.0 ± 0.2 ^b^	1365.4 ± 11.1 ^a^	97.4 ± 3.7 ^c^	55.9 ± 3.6 ^c^
	M4	30.5 ± 3.1 ^a^	64.7 ± 8.4 ^a^	39.8 ± 4.6 ^a^	5.1 ± 0.0 ^a^	12.5 ± 0.6 ^a^	0.3 ± 0.1 ^a^	84.0 ± 0.5 ^a^	1.2 ± 0.3 ^c^	14.0 ± 0.2 ^b^	1259.5 ± 21.7 ^b^	102.6 ± 6.8 ^b^	55.8 ± 5.4 ^c^
	M5	32.9 ± 1.6 ^a^	56.7 ± 3.5 ^ab^	28.6 ± 1.9 ^c^	4.8 ± 0.0 ^c^	12.9 ± 0.6 ^a^	0.3 ± 0.0 ^a^	81.1 ± 1.4 ^b^	1.7 ± 0.2 ^b^	11.8 ± 0.9 ^c^	1368.3 ± 21.3 ^a^	107.2 ± 2.0 ^b^	60.5 ± 4.8 ^b^
Yellow medium peppers	M1	10.4 ± 1.9 ^c^	35.2 ± 3.1 ^c^	33.5 ± 3.4 ^c^	6.0 ± 0.1 ^a^	2.7 ± 0.4 ^d^	0.1 ± 0.0 ^b^	92.9 ± 0.2 ^a^	0.6 ± 0.1 ^bc^	8.5 ± 0.9 ^b^	1902.2 ± 29.6 ^b^	118.3 ± 17.6 ^c^	11.6 ± 2.3 ^d^
	M2	13.5 ± 1.5 ^c^	41.6 ± 3.9 ^b^	40.6 ± 2.1 ^b^	4.9 ± 0.0 ^d^	6.3 ± 0.5 ^b^	0.4 ± 0.0 ^a^	91.4 ± 1.6 ^a^	0.7 ± 0.1 ^b^	9.4 ± 0.0 ^b^	1854.3 ± 43.3 ^b^	125.6 ± 22.2 ^c^	12.8 ± 2.8 ^d^
	M3	19.7 ± 2.2 ^b^	55.6 ± 10.1 ^a^	43.5 ± 2.3 ^b^	5.1 ± 0.0 ^c^	8.0 ± 0.8 ^a^	0.4 ± 0.1 ^a^	89.0 ± 2.0 ^a^	1.1 ± 0.1 ^a^	9.1 ± 0.4 ^b^	1773.7 ± 16.0 ^c^	106.1 ± 2.5 ^c^	17.5 ± 0.5 ^c^
	M4	22.7 ± 1.0 ^b^	65.0 ± 7.5 ^a^	41.9 ± 5.5 ^b^	5.6 ± 0.0 ^b^	6.5 ± 1.0 ^b^	0.3 ± 0.1 ^a^	92.8 ± 1.4 ^a^	0.5 ± 0.1 ^c^	12.4 ± 0.2 ^a^	1927.8 ± 0.1 ^b^	165.6 ± 4.7 ^b^	42.0 ± 0.1 ^a^
	M5	37.6 ± 5.8 ^a^	57.4 ± 6.3 ^a^	54.0 ± 6.7 ^a^	5.2 ± 0.0 ^c^	4.9 ± 0.7 ^c^	0.4 ± 0.1 ^a^	92.7 ± 0.3 ^a^	0.6 ± 0.1 ^bc^	9.0 ± 0.6 ^b^	2574.6 ± 95.1 ^a^	194.8 ± 15.7 ^a^	32.9 ± 0.7 ^b^
Orange medium peppers	M1	24.2 ± 0.6 ^d^	63.3 ± 2.6 ^b^	41.6 ± 3.4 ^b^	6.0 ± 0.1 ^a^	4.3 ± 0.5 ^c^	0.2 ± 0.0 ^b^	92.7 ± 0.4 ^a^	1.0 ± 0.0 ^b^	6.0 ± 0.4 ^c^	2149.3 ± 59.7 ^b^	164.8 ± 16.3 ^a^	25.0 ± 1.8 ^c^
	M2	27.9 ± 2.0 ^c^	71.2 ± 13.7 ^a^	41.8 ± 3.7 ^b^	5.0 ± 0.0 ^b^	5.6 ± 0.5 ^b^	0.3 ± 0.1 ^b^	93.2 ± 0.4 ^a^	0.8 ± 0.1 ^c^	9.3 ± 0.0 ^b^	2003.1 ± 29.8 ^c^	135.7 ± 2.6 ^c^	28.5 ± 3.2 ^c^
	M3	28.5 ± 1.0 ^c^	70.9 ± 13.9 ^a^	42.8 ± 4.1 ^b^	4.9 ± 0.0 ^b^	4.3 ± 0.5 ^c^	0.2 ± 0.0 ^b^	94.2 ± 0.3 ^a^	0.5 ± 0.1 ^d^	15.1 ± 0.4 ^a^	2165.3 ± 29.8 ^b^	159.0 ± 15.7 ^b^	31.9 ± 2.3 ^b^
	M4	35.8 ± 1.1 ^b^	68.2 ± 14.6 ^a^	49.6 ± 5.3 ^a^	5.0 ± 0.0 ^b^	4.8 ± 0.0 ^c^	0.3 ± 0.0 ^b^	93.1 ± 4.6 ^a^	0.7 ± 0.1 ^c^	15.3 ± 0.6 ^a^	2520.6 ± 4.8 ^a^	179.3 ± 18.6 ^a^	44.2 ± 0.3 ^a^
	M5	52.5 ± 3.4 ^a^	79.4 ± 8.7 ^a^	53.5 ± 5.0 ^a^	5.1 ± 0.0 ^b^	8.6 ± 0.4 ^a^	0.6 ± 0.0 ^a^	87.9 ± 1.7 ^a^	1.8 ± 0.0 ^a^	6.0 ± 0.3 ^c^	1994.2 ± 80.3 ^d^	163.4 ± 9.7 ^ab^	42.5 ± 1.1 ^a^
Yellow big peppers	M1	185.1 ± 12.4 ^e^	20.3 ± 1.0 ^c^	79.0 ± 0.4 ^b^	5.1 ± 0.1 ^a^	7.0 ± 0.5 ^a^	0.3 ± 0.1 ^ab^	91.7 ± 0.2 ^a^	0.9 ± 0.1 ^c^	9.3 ± 0.1 ^c^	2057.4 ± 11.6 ^a^	148.7 ± 12.2 ^a^	14.9 ± 1.2 ^d^
	M2	229.8 ± 6.2 ^d^	58.0 ± 8.9 ^b^	90.4 ± 8.4 ^a^	4.9 ± 0.0 ^a^	6.3 ± 0.0 ^b^	0.3 ± 0.1 ^ab^	91.9 ± 0.2 ^a^	1.8 ± 0.9 ^a^	17.6 ± 0.3 ^a^	1851.4 ± 26.7 ^b^	123.0 ± 13.8 ^a^	55.3 ± 9.1 ^a^
	M3	265.4 ± 20.5 ^c^	95.7 ± 16.9 ^a^	87.3 ± 0.7 ^a^	4.8 ± 0.0 ^b^	6.4 ± 0.1 ^b^	0.3 ± 0.0 ^b^	91.6 ± 0.3 ^a^	1.2 ± 0.2 ^b^	12.6 ± 0.0 ^b^	1654.5 ± 0.0 ^d^	125.7 ± 14.3 ^a^	33.7 ± 3.9 ^c^
	M4	374.4 ± 20.6 ^b^	58.0 ± 8.9 ^b^	86.2 ± 4.7 ^a^	4.8 ± 0.0 ^b^	6.6 ± 0.0 ^a^	0.2 ± 0.0 ^c^	91.3 ± 0.6 ^a^	0.8 ± 0.1 ^c^	12.0 ± 0.0 ^b^	1625.5 ± 64.8 ^d^	103.3 ± 1.8 ^b^	9.3 ± 2.1 ^e^
	M5	483.5 ± 20.7 ^a^	108.6 ± 6.1 ^a^	85.1 ± 8.7 ^a^	4.9 ± 0.0 ^a^	6.9 ± 0.3 ^a^	0.4 ± 0.0 ^a^	92.7 ± 0.1 ^a^	1.4 ± 0.2 ^b^	9.1 ± 0.1 ^c^	1710.4 ± 21.1 ^c^	103.8 ± 4.3 ^b^	40.6 ± 4.3 ^b^

Note: Values indicate Average ± Standard deviation. LD, longitudinal diameter; ED, equatorial diameter; SS, soluble solids; TA, titratable acidity; Fe, iron; K, potassium; Mg, magnesium; Na, sodium. The lowercase letters next to the deviation indicate significant differences with *p* < 0.05 between different degrees of ripeness for the same variety.

**Table 2:** Average values for the concentration of vitamin C, organic acids, total anthocyanins, capsaicin, and antioxidant activity of the pepper varieties under study.

Common Name	Maturity	Vitamin C (mg/100 g DW)	Citric Acid (mg/100 g DW)	Malic Acid (mg/100 g DW)	Tartaric Acid (mg/100 g DW)	Total Organic Acid (mg/100 g DW)	Total Anthocyanins (mg C-3-gl/100 g DW)	Capsaicin (mg/100 g DW)	Antioxidant Activity (mmol TE/100 g DW)	
									DPPH	ABTS
Malagueta chili	M1	17.1 ± 0.8 ^e^	175.0 ± 5.9 ^a^	311.1 ± 14.9 ^b^	83.9 ± 2.1 ^c^	570.0 ± 18.7 ^b^	13.7 ± 2.1 ^a^	853.3 ± 15.4 ^c^	3.9 ± 0.2 ^a^	3.0 ± 0.4 ^b^
	M2	26.1 ± 1.1 ^d^	141.3 ± 0.9 ^b^	270.5 ± 18.2 ^c^	95.2 ± 3.3 ^b^	507.0 ± 20.6 ^c^	17.5 ± 3.3 ^a^	915.8 ± 30.8 ^b^	3.2 ± 0.4 ^b^	3.1 ± 0.4 ^b^
	M3	66.9 ± 3.9 ^c^	68.6 ± 3.5 ^e^	234.9 ± 7.6 ^d^	54.3 ± 3.0 ^d^	357.8 ± 14.1 ^d^	nd	695.5 ± 27.2 ^e^	2.2 ± 0.1 ^c^	2.8 ± 0.4 ^b^
	M4	149.0 ± 0.8 ^b^	84.9 ± 4.5 ^d^	359.8 ± 34.9 ^b^	74.0 ± 5.8 ^c^	518.6 ± 45.2 ^c^	nd	813.4 ± 18.1 ^d^	2.9 ± 0.1 ^b^	2.9 ± 0.4 ^b^
	M5	235.4 ± 2.9 ^a^	133.4 ± 6.5 ^c^	587.6 ± 8.5 ^a^	124.7 ± 6.0 ^a^	845.6 ± 21.0 ^a^	nd	1949.8 ± 91.3 ^a^	3.4 ± 0.0 ^b^	3.7 ± 0.5 ^a^
Habanero chili	M1	5631.9 ± 27.3 ^c^	1346.5 ± 12.4 ^c^	479.6 ± 48.2 ^bc^	438.4 ± 19.6 ^c^	2264.5 ± 19.2 ^c^	27.2 ± 3.3 ^b^	354.8 ± 70.1 ^a^	39.9 ± 3.3 ^a^	5.1 ± 0.5 ^c^
	M2	9027.7 ± 40.9 ^b^	2252.6 ± 28.7 ^a^	561.5 ± 11.5 ^a^	670.1 ± 36.3 ^b^	3484.2 ± 76.7 ^a^	19.9 ± 2.6 ^c^	363.2 ± 25.9 ^a^	41.3 ± 0.6 ^a^	6.8 ± 0.3 ^a^
	M3	10,319.5 ± 29.9 ^a^	1038.5 ± 10.9 ^e^	450.0 ± 31.2 ^c^	352.4 ± 27.1 ^a^	1814.0 ± 16.8 ^e^	21.3 ± 3.7 ^c^	171.2 ± 32.1 ^c^	39.1 ± 2.3 ^a^	6.0 ± 0.4 ^b^
	M4	5432.8 ± 44.7 ^d^	1490.0 ± 10.3 ^b^	499.9 ± 28.6 ^b^	341.6 ± 16.5 ^d^	2331.6 ± 14.9 ^b^	9.1 ± 0.8 ^d^	251.9 ± 25.4 ^b^	3.7 ± 0.3 ^b^	6.1 ± 0.2 ^b^
	M5	4635.0 ± 12.3 ^e^	1295.3 ± 46.0 ^d^	411.4 ± 1.5 ^d^	248.3 ± 1.7 ^e^	1955.0 ± 46.2 ^d^	61.0 ± 6.5 ^a^	165.5 ± 19.3 ^c^	3.0 ± 0.1 ^c^	5.6 ± 0.5 ^c^
Jalapeño chili	M1	453.9 ± 33.1	323.5 ± 4.1 ^e^	1164.3 ± 67.8 ^a^	14.5 ± 0.5 ^e^	1502.3 ± 63.2 ^e^	15.6 ± 2.2 ^b^	nd	4.7 ± 0.1 ^b^	3.1 ± 0.2 ^d^
	M2	3484.3 ± 20.1 ^b^	1507.0 ± 14.7 ^c^	799.2 ± 29.7 ^b^	2244.8 ± 52.2 ^b^	4551.1 ± 13.9 ^c^	18.7 ± 4.0 ^b^	285.4 ± 2.9 ^b^	38.9 ± 3.7 ^a^	4.1 ± 0.4 ^c^
	M3	1004.7 ± 3.8 ^d^	844.9 ± 9.0 ^d^	855.5 ± 57.1 ^b^	378.4 ± 2.7 ^d^	2078.8 ± 68.8 ^d^	18.3 ± 2.4 ^b^	88.5 ± 10.0 ^d^	2.3 ± 0.1 ^d^	4.4 ± 0.4 ^c^
	M4	3303.6 ± 56.2 ^c^	2415.6 ± 27.7 ^a^	880.8 ± 68.4 ^b^	1807.8 ± 45.7 ^c^	5104.2 ± 39.1 ^a^	31.2 ± 3.9 ^a^	178.4 ± 20.0 ^c^	3.7 ± 0.1 ^c^	4.9 ± 0.4 ^b^
	M5	4326.6 ± 72.7 ^a^	2173.1 ± 28.1 ^b^	842.3 ± 85.9 ^b^	2673.4 ± 15.8 ^a^	4638.8 ± 14.3 ^b^	30.5 ± 4.1 ^a^	353.2 ± 16.3 ^a^	4.0 ± 0.3 ^c^	5.9 ± 0.5 ^a^
Orange cherry peppers	M1	1023.3 ± 53.1 ^a^	517.0 ± 7.5 ^a^	2972.2 ± 42.5 ^a^	51.8 ± 0.9 ^d^	3541.0 ± 50.9 ^a^	23.6 ± 3.7 ^b^	nd	5.2 ± 0.5 ^a^	2.0 ± 0.4 ^a^
	M2	889.6 ± 2.2 ^b^	436.5 ± 1.0 ^b^	3015.7 ± 15.3 ^a^	89.6 ± 30.4 ^b^	3541.7 ± 16.1 ^a^	13.6 ± 1.9 ^c^	nd	4.5 ± 0.3 ^bc^	1.9 ± 0.1 ^a^
	M3	799.8 ± 26.2 ^c^	435.4 ± 0.3 ^b^	2245.5 ± 20.1 ^c^	104.5 ± 15.6 ^a^	2785.5 ± 18.6 ^c^	20.6 ± 3.9 ^b^	nd	4.7 ± 0.3 ^b^	2.0 ± 0.2 ^a^
	M4	883.9 ± 5.8 ^b^	356.9 ± 7.6 ^c^	2648.6 ± 13.8 ^b^	68.9 ± 0.6 ^c^	3074.5 ± 22.0 ^b^	32.0 ± 4.8 ^a^	nd	5.2 ± 0.4 ^a^	1.5 ± 0.1 ^b^
	M5	647.9 ± 33.2 ^d^	373.9 ± 26.6 ^c^	2285.4 ± 14.5 ^c^	69.9 ± 1.6 ^c^	729.2 ± 10.5 ^d^	21.5 ± 2.4 ^b^	nd	4.2 ± 0.6 ^c^	1.4 ± 0.5 ^b^
Yellow medium peppers	M1	11,399.9 ± 48.1 ^a^	410.8 ± 5.2 ^e^	3276.3 ± 87.5 ^b^	131.7 ± 1.5 ^b^	3818.8 ± 94.3 ^b^	17.1 ± 1.3 ^b^	nd	5.0 ± 0.5 ^b^	2.0 ± 0.0 ^a^
	M2	517.9 ± 13.1 ^e^	551.4 ± 17.7 ^c^	1667.1 ± 11.2 ^e^	158.4 ± 19.0 ^a^	2376.9 ± 14.9 ^e^	9.1 ± 0.2 ^c^	nd	6.0 ± 0.1 ^a^	2.1 ± 0.4 ^a^
	M3	969.0 ± 58.2 ^c^	732.2 ± 46.5 ^a^	2719.8 ± 11.6 ^c^	169.0 ± 7.3 ^a^	3621.0 ± 76.8 ^c^	11.0 ± 3.3 ^c^	nd	5.5 ± 0.1 ^b^	1.7 ± 0.4 ^a^
	M4	1949.5 ± 70.2 ^b^	465.2 ± 14.3 ^d^	4740.4 ± 20.7 ^a^	92.5 ± 2.4 ^c^	5298.1 ± 22.3 ^a^	25.7 ± 4.4 ^a^	nd	6.2 ± 0.0 ^a^	1.5 ± 0.2 ^a^
	M5	649.5 ± 33.5 ^d^	652.5 ± 42.2 ^b^	2040.1 ± 1.9 ^d^	133.6 ± 1.7 ^b^	2826.2 ± 42.0 ^d^	19.3 ± 5.1 ^a^	nd	5.7 ± 0.5 ^a^	1.3 ± 0.1 ^b^
Orange medium peppers	M1	28.3 ± 0.5 ^d^	273.1 ± 61.1 ^c^	513.5 ± 0.9 ^e^	127.7 ± 1.7 ^a^	914.3 ± 58.4 ^e^	18.7 ± 2.3 ^c^	nd	2.8 ± 0.1 ^c^	1.1 ± 0.1 ^b^
	M2	962.1 ± 48.3 ^a^	441.7 ± 6.4 ^b^	2951.9 ± 14.9 ^a^	73.6 ± 3.4 ^c^	3467.2 ± 15.9 ^a^	25.7 ± 4.9 ^c^	nd	5.7 ± 0.5 ^a^	1.8 ± 0.2 ^b^
	M3	146.1 ± 7.8 ^c^	640.3 ± 57.0 ^a^	1078.0 ± 75.7 ^d^	78.9 ± 7.3 ^c^	1797.2 ± 13.9 ^d^	187.1 ± 1.8 ^b^	nd	5.4 ± 0.3 ^a^	2.4 ± 0.5 ^a^
	M4	551.9 ± 14.1 ^b^	679.4 ± 44.0 ^a^	2279.9 ± 16.5 ^b^	96.6 ± 12.7 ^b^	3055.9 ± 22.2 ^b^	367.5 ± 2.9 ^a^	nd	4.4 ± 0.1 ^b^	1.8 ± 0.3 ^b^
	M5	575.2 ± 46.6 ^b^	585.6 ± 65.2 ^a^	2013.7 ± 29.7 ^c^	104.7 ± 12.9 ^ab^	2704.0 ± 37.6 ^c^	0.6 ± 0.1 ^d^	nd	3.9 ± 0.4 ^b^	2.0 ± 0.3 ^b^
Yellow big peppers	M1	1580.7 ± 10.5 ^a^	520.6 ± 9.2 ^d^	4178.9 ± 31.0 ^a^	106.1 ± 0.8 ^b^	4805.6 ± 41.0 ^a^	10.0 ± 1.4 ^a^	nd	5.4 ± 0.3 ^a^	2.4 ± 0.2 ^b^
	M2	1581.1 ± 10.3 ^a^	593.3 ± 2.8 ^c^	3941.8 ± 45.9 ^b^	127.8 ± 7.9 ^a^	4662.9 ± 51.0 ^b^	8.7 ± 2.2 ^a^	nd	5.6 ± 0.7 ^a^	2.4 ± 0.1 ^b^
	M3	1349.3 ± 85.4 ^b^	640.6 ± 9.8 ^a^	3669.4 ± 14.9 ^c^	94.5 ± 8.3 ^c^	4404.5 ± 16.7 ^c^	5.2 ± 1.2 ^c^	nd	5.3 ± 0.1 ^a^	2.9 ± 0.2 ^a^
	M4	1342.3 ± 59.2 ^b^	621.7 ± 6.7 ^b^	3498.5 ± 85.9 ^d^	81.0 ± 4.3 ^d^	4201.2 ± 93.2 ^d^	5.9 ± 1.4 ^c^	nd	5.5 ± 0.2 ^a^	2.8 ± 0.2 ^a^
	M5	1335.3 ± 32.9 ^b^	602.8 ± 3.7 ^c^	3327.7 ± 22.8 ^e^	67.5 ± 0.2 ^e^	3998.0 ± 19.3 ^e^	6.5 ± 1.6 ^b^	nd	5.6 ± 0.2 ^a^	2.7 ± 0.1 ^a^

Note: Values indicate Average ± Standard deviation—C-3-gl; Cyanidin-3-glucoside; TE, Trolox equivalent; nd, No detectable limit. The lowercase letters next to the deviation indicate significant differences with *p* < 0.05 between different degrees of ripeness for the same variety.

**Table 3:** Average values for the concentration of carotenoids of the pepper varieties under study.

Common Name	Maturity	α-Carotene (mg/100 g DW)	β-Carotene (mg/100 g DW)	Cantaxanthin (mg/100 g DW)	Astaxanthin (mg/100 g DW)	Capxanthin (mg/100 g DW)	β-Cryptoxanthin (mg/100 g DW)	Violaxanthin (mg/100 g DW)	Luteoxanthin (mg/100 g DW)	Zeaxanthin (mg/100 g DW)	Lutein (mg/100 g DW)	Zeinoxanthin (mg/100 g DW)	Total Carotenoids (mg/100 g DW)
Malagueta chili	M1							0.7 ± 0.1 ^a^		0.2 ± 0.0 ^c^	4.9 ± 0.2 ^a^	0.2 ± 0.0 ^a^	5.9 ± 0.0 ^d^
	M2	0.6 ± 0.1 ^d^	1.2 ± 0.0 ^d^					0.7 ± 0.1 ^a^		0.2 ± 0.0 ^c^	4.2 ± 0.0 ^b^	0.1 ± 0.0 ^b^	7 ± 0.2 ^c^
	M3	5.7 ± 0.4 ^c^	48.6 ± 1.2 ^c^	5.8 ± 0.0 ^c^	96.2 ± 4.2 ^b^	427.3 ± 10.7 ^c^	0.7 ± 0.1 ^b^			0.7 ± 0.0 ^b^			585 ± 11.2 ^b^
	M4	16.3 ± 0.0 ^a^	82.6 ± 0.2 ^b^	9.2 ± 0.0 ^b^	64.9 ± 0.2 ^c^	542.1 ± 1.9 ^b^	0.7 ± 0.0 ^b^			0.8 ± 0.0 ^b^			716.7 ± 2.0 ^b^
	M5	7.2 ± 0.0 ^b^	183.1 ± 0.0 ^a^	17.8 ± 0.0 ^a^	211.6 ± 0.0 ^a^	1373.3 ± 0.0 ^a^	1.8 ± 0.0 ^a^			1.3 ± 0.0 ^a^			1796.1 ± 0.0 ^a^
Habanero chili	M1	1.3 ± 0.4 ^e^								0.6 ± 0.1 ^b^	8.4 ± 2.0 ^a^		10.2 ± 2.6 ^e^
	M2	3.5 ± 0.2 ^d^	69.9 ± 0.2 ^c^				0.7 ± 0.1 ^d^			0.2 ± 0.0 ^c^	7.8 ± 0.5 ^a^	0.4 ± 0.0 ^b^	82.4 ± 7.9 ^d^
	M3	59.3 ± 5.5 ^b^	383.1 ± 3.5 ^a^	303.2 ± 17.4 ^a^	47.8 ± 3.8 ^a^	262.5 ± 24.2 ^a^	5 ± 1.9 ^a^			0.8 ± 0.1 ^b^	5 ± 0.7 ^b^		1105.8 ± 39.8 ^a^
	M4	79.9 ± 5.9 ^a^	89.6 ± 0.8 ^b^	208.5 ± 25.6 ^b^	32.3 ± 3.2 ^b^	199.6 ± 12.7 ^b^	4.3 ± 0.1 ^b^			1.3 ± 0.3 ^a^	4.1 ± 0.1 ^b^		683 ± 63.6 ^b^
	M5	13 ± 0.3 ^c^	62.1 ± 0.7 ^c^	159.1 ± 5.6 ^c^	49.8 ± 1.9 ^a^	34.8 ± 3.7 ^c^	1.2 ± 0.1 ^c^			0.6 ± 0.1 ^b^		1.2 ± 0.0 ^a^	327.3 ± 2.9 ^c^
Jalapeño chili	M1							3.5 ± 0.0 ^c^		0.8 ± 0.0 ^b^	16.2 ± 0.1 ^a^	0.4 ± 0.0 ^a^	20.9 ± 0.1 ^e^
	M2	3.4 ± 0.0 ^c^	7.3 ± 0.1 ^b^		29.2 ± 0.3 ^d^		0.8 ± 0.0 ^b^	4.5 ± 0.0 ^b^		1.3 0.2 ^a^	10.1 ± 0.1 ^b^		57.1 ± 0.6 ^d^
	M3	3.1 ± 0.2 ^c^	7.3 ± 0.8 ^b^	26.8 ± 0.4 ^c^	75.7 ± 4.7 ^c^	25.7 ± 1.2 ^c^	0.2 ± 0.0 ^c^	1.7 ± 0.3 ^d^		0.2 ± 0.1 ^c^	2.4 ± 1.1 ^cd^	0.2 ± 0.1 ^b^	137.4 ± 3.6 ^c^
	M4	5.9 ± 0.2 ^b^	8.5 ± 1.7 ^b^	82.1 ± 18.4 ^b^	149.9 ± 3.0 ^b^	58.4 ± 10.2 ^b^	0.8 ± 0.2 ^b^	3.6 ± 0.2 ^c^			1.2 ± 0.0 ^d^		310.3 ± 29.0 ^b^
	M5	12.6 ± 0.6 ^a^	33.4 ± 15.4 ^a^	369.4 ± 82.9 ^a^	194.7 ± 6.9 ^a^	83.1 ± 19.4 ^a^	1.1 ± 0.4 ^a^	8.1 ± 3.5 ^a^			3.5 ± 1.4 ^c^		705.8 ± 19.3 ^a^
Orange cherry peppers	M1	142.8 ± 2.7 ^a^	245.2 ± 5.1 ^c^				35.4 ± 3.6 ^a^	11.2 ± 0.7 ^a^	16.9 ± 0.9 ^a^	1.8 ± 0.0 ^c^	42.7 ± 3.8 ^a^		604.3 ± 25.3 ^c^
	M2	71.5 ± 1.8 ^b^	343.8 ± 8.6 ^b^				6.2 ± 0.2 ^b^	3.8 ± 0.1 ^c^	7.9 ± 0.2 ^b^	1.3 ± 0.0 ^d^	20.8 ± 0.5 ^b^		510 ± 10.8 ^d^
	M3	149.6 ± 0.2 ^a^	511.7 ± 2.9 ^a^				32.4 ± 0.1 ^a^	4.2 ± 0.0 ^b^		22.9 ± 0.1 ^a^	39.8 ± 0.4 ^a^	2.7 ± 0	763.4 ± 3.6 ^b^
	M4	37.2 ± 3.1 ^c^	89.3 ± 1.7 ^d^	193.3 ± 9.7 ^a^		713.6 ± 26.1 ^a^	4.4 ± 0.2 ^c^	4.1 ± 0.0 ^b^	3.5 ± 0.5 ^c^	3.8 ± 0.3 ^b^	12.9 ± 0.6 ^c^		1062.1 ± 35.1 ^a^
	M5	10 ± 0.2 ^d^	40.8 ± 0.7 ^e^	79.5 ± 0.8 ^b^		505.1 ± 4.6 ^b^	2.4 ± 0.0 ^d^	1.5 ± 0.1 ^d^	1 ± 0.1 ^d^	1.1 ± 0.1 ^d^	1.4 ± 0.2 ^d^		642.8 ± 3.4 ^c^
Yellow medium peppers	M1							0.5 ± 0.0 ^e^		1.6 ± 0.1 ^d^	24 ± 0.9 ^b^		27.2 ± 0.9 ^d^
	M2	8.9 ± 0.6 ^b^	7.4 ± 0.8 ^d^					17.1 ± 0.9 ^b^	2.4 ± 0.2 ^c^	12 ± 0.5 ^b^	48.1 ± 4.6 ^a^		97.9 ± 4.4 ^c^
	M3	45.3 ± 0.2 ^a^	1174.7 ± 13.3 ^a^				8.6 ± 0.7 ^a^	3.8 ± 0.3 ^d^		39.5 ± 1.5 ^a^	25.5 ± 0.0 ^b^	3.5 ± 0.2 ^a^	1300.9 ± 12.8 ^a^
	M4	29.1 ± 1.6 ^b^	128.5 ± 12.5 ^b^				6.1 ± 1.1 ^b^	23 ± 1.8 ^a^	68.5 ± 6.1 ^a^	6.2 ± 0.4 ^c^	55.4 ± 7.7 ^a^	2.7 ± 0.1 ^b^	319.4 ± 31.4 ^b^
	M5	5.7 ± 0.5 ^c^	40.2 ± 1.1 ^c^				1.5 ± 0.2 ^c^	5.1 ± 0.4 ^c^	8.6 ± 0.6 ^b^	1.2 ± 0.1 ^e^	27.5 ± 2.4 ^b^	0.7 ± 0.1 ^c^	90.6 ± 3.2 ^c^
Orange medium peppers	M1		40 ± 1.3 ^c^					4.1 ± 0.2 ^d^		1.7 ± 0.1 ^c^	52.2 ± 2.9 ^a^		98 ± 4.5 ^e^
	M2	7.2 ± 0.4 ^c^	16.7 ± 3.4 ^d^	58 ± 2.8 ^c^		300.9 ± 2.3 ^c^	1.8 ± 0.1 ^d^	8.5 ± 0.2 ^c^	3.5 ± 0.2	3.7 ± 0.0 ^a^	31.2 ± 0.3 ^b^		431.5 ± 7.5 ^c^
	M3	6.7 ± 0.8 ^c^	10.6 ± 0.2 ^d^	63.6 ± 1.1 ^c^		198.9 ± 6.2 ^d^	2.7 ± 0.0 ^c^	1.5 ± 0.0 ^e^		2.3 ± 0.0 ^b^	7.5 ± 0.0 ^d^		293.8 ± 5.2 ^d^
	M4	50.1 ± 2.0 ^b^	393.8 ± 0.2 ^b^	344.9 ± 5.0 ^b^		2192.4 ± 33.8 ^b^	20.7 ± 0.7 ^b^	22.6 ± 0.2 ^b^		2 ± 0.2 ^b^	14.9 ± 0.3 ^c^		3132.4 ± 34.7 ^b^
	M5	144.7 ± 13.1 ^a^	1979.9 ± 23.2 ^a^	1048.4 ± 88.3 ^a^		5679.1 ± 44.5 ^a^	69.1 ± 5.1 ^a^	79 ± 5.6 ^a^		1.5 ± 0.2 ^c^	34.5 ± 2.0 ^b^		9495.8 ± 33.4 ^a^
Yellow big peppers	M1	41.2 ± 0.0 ^a^	248.7 ± 8.3 ^a^				11 ± 0.5 ^a^	24.7 ± 1.1 ^a^		4.5 ± 0.4	65.7 ± 1.3 ^a^	2.8 ± 0.4 ^a^	397.7 ± 11.3 ^a^
	M2	11.7 ± 1.1 ^b^	35.6 ± 2.3 ^b^				1.7 ± 0.1 ^c^	3.8 ± 0.3 ^b^			7.6 ± 0.3 ^b^	0.7 ± 0.0 ^b^	61.2 ± 4.0 ^b^
	M3	8.7 ± 0.3 ^c^	24.5 ± 0.2 ^c^				2 ± 0.0 ^b^	2.6 ± 0.2 ^c^			8.5 ± 0.4 ^b^	0.3 ± 0.1 ^c^	46.7 ± 0.6 ^c^
	M4	7.4 ± 0.2 ^d^	19.3 ± 1.1 ^d^				1.6 ± 0.0 ^c^	2 ± 0.1 ^d^			6.7 ± 0.3 ^c^	0.2 ± 0.0 ^c^	37.1 ± 1.1 ^d^
	M5	6 ± 0.0 ^e^	14.2 ± 1.9 ^e^				1.2 ± 0.1 ^d^	1.3 ± 0.1 ^e^			4.8 ± 0.1 ^d^	0.2 ± 0.0 ^c^	27.6 ± 1.6 ^e^

Note: Values indicate Average ± Standard deviation. Lowercase letters next to the deviation indicate significant differences with *p* < 0.05 between different degrees of ripeness for the same variety. Several cells indicate the absence of the compound.

**Table 4:** Average values for the concentration of phenolics of the pepper varieties under study.

Common Name	Maturity	Gallic Acid (mg/100 g DW)	4-Hydroxybenzoic Acid (mg/100 g DW)	Catechin (mg/100 g DW)	Vanillic Acid (mg/100 g DW)	*m*-Coumaric Acid (mg/100 g DW)	Syringic Acid (mg/100 g DW)	Chlorogenic Acid (mg/100 g DW)	Caffeic Acid (mg/100 g DW)	Naringenin (mg/100 g DW)	Ferulic Acid (mg/100 g DW)	Sinapic Acid (mg/100 g DW)	Kaempferol (mg/100 g DW)	Quercetin Glucoside (mg/100 g DW)	Quercetin (mg/100 g DW)	Total Phenolics (mg/100 g DW)
Malagueta chili	M1	70.7 ± 5.8 ^a^	25.2 ± 1.3 ^b^	6.2 ± 0.1 ^d^	6.5 ± 0.3 ^e^	97.6 ± 0.5 ^b^	11.3 ± 1.8 ^d^	14 ± 0.2 ^c^	27.4 ± 0.9 ^d^	275.7 ± 0.7 ^d^	37.3 ± 4.9 ^d^		22.9 ± 0.5 ^b^	19.7 ± 1.3 ^b^	13.8 ± 0.2 ^c^	628.2 ± 11.5 ^d^
	M2	62.2 ± 1.6 ^b^	41.7 ± 0.4 ^a^	28.9 ± 0.1 ^a^	9.2 ± 1.4 ^d^	109.9 ± 1.7 ^a^	15.1 ± 0.5 ^c^	24.4 ± 2.5 ^a^	32.9 ± 0.2 ^c^	496 ± 6.6 ^a^	34.9 ± 1.4 ^d^		25.4 ± 0.1 ^a^	20.7 ± 3.0 ^b^	13.6 ± 0.3 ^c^	915 ± 6.4 ^b^
	M3	13.4 ± 0.3 ^e^	7.6 ± 0.1 ^c^	20.3 ± 0.1 ^b^	23.3 ± 0.1 ^c^	40.9 ± 0.0 ^d^	28 ± 0.4 ^b^	8.4 ± 0.1 ^d^	34.5 ± 1.1 ^c^	300.4 ± 0.2 ^c^	49 ± 0.3 ^c^		10.2 ± 0.1 ^d^	33.5 ± 0.3 ^a^	21.7 ± 0.3 ^a^	591.3 ± 0.7 ^e^
	M4	17.5 ± 0.2 ^d^	7 ± 0.1 ^d^	26 ± 0.0 ^a^	34 ± 0.1 ^a^	46.7 ± 3.5 ^c^	40.4 ± 1.2 ^a^	6.7 ± 0.3 ^e^	104.5 ± 4.2 ^a^	473 ± 0.2 ^b^	169.1 ± 5.6 ^a^		12.1 ± 0.1 ^c^	30.8 ± 4.8 ^a^	23.3 ± 5.8 ^a^	991.1 ± 4.1 ^a^
	M5	20.3 ± 2.6 ^c^	4.4 ± 0.1 ^e^	18.9 ± 0.1 ^c^	26.5 ± 0.3 ^b^	31.1 ± 0.7 ^e^	28.6 ± 0.2 ^b^	16.1 ± 0.5 ^b^	65.5 ± 3.6 ^b^	488.8 ± 5.5 ^a^	134.3 ± 12.4 ^b^		10.3 ± 0.2 ^d^	19.1 ± 0.1 ^b^	15.9 ± 0.1 ^b^	879.9 ± 7.5 ^c^
Habanero chili	M1	5.7 ± 0.0 ^d^	131.6 ± 4.4 ^a^			317.6 ± 13.1 ^a^	22.6 ± 0.5 ^a^		168.4 ± 14.4 ^ab^					188 ± 3.1 ^a^	66.1 ± 2.6 ^a^	899.9 ± 26.7 ^a^
	M2	10.6 ± 0.9 ^c^	128.5 ± 3.0 ^b^			134.7 ± 6.6 ^c^	12.1 ± 1.4 ^b^		179.8 ± 5.1 ^a^					80.3 ± 4.5 ^c^	49 ± 5.2 ^b^	595 ± 9.9 ^b^
	M3	12.4 ± 0.2 ^b^	112.6 ± 2.8 ^c^			165 ± 8.5 ^b^	6.1 ± 0.1 ^d^		123.1 ± 4.5 ^c^					117.4 ± 6.5 ^b^	38.2 ± 1.7 ^c^	574.8 ± 24.3 ^b^
	M4	12.7 ± 0.8 ^ab^	97.6 ± 2.2 ^d^			123.5 ± 7.0 ^c^	10.3 ± 2.2 ^c^	41.6 ± 0.6 ^a^	167.1 ± 6.3 ^ab^					88.8 ± 4.1 ^c^	40.2 ± 1.9 ^c^	581.8 ± 25.1 ^b^
	M5	14.1 ± 0.6 ^a^	60.7 ± 3.1 ^e^			103.2 ± 6.3 ^d^	21.5 ± 1.2 ^a^	32.7 ± 2.6 ^b^	152.8 ± 10.6 ^b^					63.4 ± 0.8 ^d^	43.5 ± 4.2 ^bc^	492 ± 25.8 ^c^
Jalapeño chili	M1	90.3 ± 3.2 ^a^	8.5 ± 0.8 ^e^	7.9 ± 1.3 ^c^	1.1 ± 0.0 ^d^	55.5 ± 0.4 ^a^	15.6 ± 0.2 ^b^	12.4 ± 0.2 ^a^	25.2 ± 0.6 ^c^	25.1 ± 0.8 ^a^	60.2 ± 0.2 ^a^		17.8 ± 0.1 ^b^	17.3 ± 0.9 ^c^	7.8 ± 0.1 ^b^	344.6 ± 3.3 ^a^
	M2	7.7 ± 0.3 ^c^	69.8 ± 5.2 ^c^	4.1 ± 0.1 ^d^	2.5 ± 0.1 ^a^	46.8 ± 2.0 ^b^	4.9 ± 0.1 ^d^	13.3 ± 0.5 ^a^	29.3 ± 0.6 ^b^				30.8 ± 2.5 ^a^	19.9 ± 1.5 ^b^	10.2 ± 0.9 ^a^	239.2 ± 8.7 ^d^
	M3	88.1 ± 0.0 ^a^	24.8 ± 0.2 ^d^	9.8 ± 0.1 ^b^	1.7 ± 0.0 ^c^	55.9 ± 0.3 ^a^	19.4 ± 0.2 ^a^	5.8 ± 0.0 ^d^	40.1 ± 0.3 ^a^	13.3 ± 0.2 ^b^	36.3 ± 1.6 ^b^		17.5 ± 0.0 ^b^	14.7 ± 0.2 ^d^	9.7 ± 0.1 ^a^	327.2 ± 1.5 ^b^
	M4	9.6 ± 0.7 ^bc^	95.3 ± 0.4 ^b^	7.6 ± 0.4 ^c^	1.9 ± 0.1 ^b^	30.5 ± 2.3 ^c^	3.5 ± 0.1 ^e^	11.3 ± 1.1 ^ab^	42.2 ± 2.6 ^a^				26.8 ± 3.0 ^a^	28.4 ± 1.5 ^a^	8.8 ± 0.9 ^a^	265.7 ± 8.4 ^c^
	M5	11.6 ± 3.0 ^b^	119.7 ± 0.6 ^a^	12.6 ± 0.0 ^a^	1.8 ± 0.1 ^bc^	44.3 ± 1.5 ^b^	5.7 ± 0.1 ^c^	7 ± 0.2 ^c^	22.8 ± 5.2 ^c^				17.1 ± 2.8 ^b^	2.9 ± 0.2 ^e^	2.2 ± 0.1 ^c^	247.7 ± 12.4 ^c^
Orange cherry peppers	M1	156.3 ± 16.6 ^a^			2.4 ± 0.3 ^d^		34.1 ± 3.5 ^a^	14.1 ± 1.2 ^d^	79.4 ± 10.6 ^d^		54.2 ± 3.3 ^d^	16.2 ± 1.0 ^d^	2.3 ± 0.1 ^b^	16.7 ± 1.8 ^b^	8.4 ± 0.5 ^c^	384.1 ± 6.6 ^d^
	M2	159 ± 1.5 ^a^			10.4 ± 0.9 ^b^		19.6 ± 1.7 ^d^	29.7 ± 1.1 ^b^	132.9 ± 23.0 ^c^		97.3 ± 7.7 ^b^	19.5 ± 0.4 ^c^	2.5 ± 0.4 ^b^	39.3 ± 4.9 ^a^	15.1 ± 0.6 ^a^	525.2 ± 7.9 ^b^
	M3	134 ± 2.2 ^b^			9.5 ± 0.0 ^c^		24.1 ± 0.3 ^c^	40.7 ± 2.8 ^a^	225.7 ± 11.3 ^a^		116.9 ± 7.5 ^a^	15 ± 2.5 ^d^	2.3 ± 0.0 ^b^	18.2 ± 1.0 ^b^	9.7 ± 0.4 ^c^	596 ± 3.7 ^a^
	M4	145.1 ± 0.9 ^a^			15.1 ± 0.4 ^a^		33.1 ± 0.5 ^a^	26.6 ± 0.4 ^c^	155.6 ± 3.1 ^c^		78.9 ± 4.4 ^c^	42 ± 1.2 ^a^	2.2 ± 0.1 ^b^	6.9 ± 0.2 ^d^	11.5 ± 0.2 ^b^	517 ± 9.5 ^b^
	M5	115.5 ± 1.5 ^c^			8.4 ± 1.3 ^c^		28.2 ± 2.8 ^b^	27.9 ± 1.2 ^bc^	169.8 ± 2.2 ^b^		83 ± 4.6 ^c^	26.6 ± 0.7 ^b^	3.3 ± 0.2 ^a^	11.9 ± 1.7 ^c^	11.9 ± 1.8 ^b^	486.6 ± 4.8 ^c^
Yellow medium peppers	M1	205.7 ± 18.9 ^a^					63.2 ± 5.0 ^c^	79.3 ± 10.2 ^a^					14 ± 1.4 ^b^	109.9 ± 10.4 ^a^	115.9 ± 7.8 ^a^	587.9 ± 5.4 ^a^
	M2	27.6 ± 3.0 ^d^					52.5 ± 7.9 ^c^	43.1 ± 2.6 ^b^				24.7 ± 2.4 ^a^		55 ± 5.3 ^b^	48.9 ± 4.5 ^b^	251.9 ± 2.6 ^d^
	M3	125.1 ± 5.7 ^c^					21.9 ± 0.9 ^d^	12.4 ± 0.4 ^c^				17.3 ± 0.3 ^b^	2.9 ± 0.3 ^c^	7.6 ± 0.8 ^c^	10.2 ± 0.2 ^c^	197.4 ± 4.8 ^e^
	M4	143.4 ± 13.8 ^b^					101.6 ± 2.4 ^b^	46.1 ± 2.5 ^b^				10.6 ± 1.1 ^d^	21.7 ± 2.4 ^a^	116.8 ± 14.2 ^a^	107.2 ± 11.2 ^a^	547.6 ± 10.7 ^b^
	M5	25.3 ± 0.5 ^d^					156.1 ± 15.1 ^a^	85 ± 4.6 ^a^				14.8 ± 1.3 ^c^	19 ± 2.1 ^a^	47.9 ± 4.7 ^b^	51.4 ± 4.9 ^b^	399.5 ± 34.7 ^c^
Orange medium peppers	M1	37.1 ± 3.4 ^c^					29.3 ± 0.4 ^e^	61.5 ± 1.5 ^b^					7.9 ± 0.4 ^c^	103.4 ± 2.8 ^b^	100.4 ± 2.9 ^c^	339.7 ± 9.7 ^d^
	M2	52.6 ± 0.4 ^a^					85.4 ± 2.4 ^d^	75.1 ± 3.7 ^a^					29.3 ± 7.0 ^a^	127.8 ± 3.6 ^a^	149.9 ± 11.6 ^a^	520.2 ± 28.0 ^a^
	M3	42.5 ± 1.6 ^b^					128.1 ± 1.9 ^b^	50.8 ± 1.5 ^c^					31.7 ± 0.3 ^a^	90.6 ± 1.6 ^c^	117.9 ± 3.8 ^b^	461.6 ± 28.3 ^b^
	M4	22.7 ± 0.2 ^d^					136.3 ± 4.0 ^a^	43 ± 0.4 ^d^					32.8 ± 0.5 ^a^	64.6 ± 0.2 ^d^	78.5 ± 0.1 ^d^	377.9 ± 5.2 ^c^
	M5	20.3 ± 0.7 ^d^					113.5 ± 1.7 ^c^	41.7 ± 0.4 ^e^					17.3 ± 1.1 ^b^	32.6 ± 7.5 ^e^	40.9 ± 1.3 ^e^	266.2 ± 12.7 ^e^
Yellow big peppers	M1	178.5 ± 21.8 ^c^					24.1 ± 2.5 ^c^	19.1 ± 2.0 ^ab^				19.7 ± 1.3 ^d^	3.2 ± 0.3 ^b^	15.1 ± 1.7 ^c^	12.7 ± 1.5 ^b^	272.4 ± 31.1 ^d^
	M2	228.3 ± 5.5 ^b^					40 ± 1.0 ^a^	22.4 ± 2.5 ^a^				36.5 ± 1.5 ^a^	1.1 ± 0.0 ^d^	33.4 ± 6.7 ^a^	19 ± 3.0 ^a^	380.8 ± 18.2 ^b^
	M3	275.9 ± 11.7 ^a^					45.4 ± 4.0 ^a^	22.2 ± 1.7 ^a^				40.8 ± 4.8 ^a^	3.7 ± 0.3 ^a^	21.3 ± 2.4 ^b^	20.2 ± 0.7 ^a^	429.4 ± 15.4 ^a^
	M4	213 ± 9.7 ^b^					36.5 ± 2.2 ^b^	17.4 ± 1.0 ^b^				31.2 ± 2.4 ^b^	2.4 ± 0.2 ^c^	18.4 ± 2.6 ^bc^	14.3 ± 0.9 ^b^	333.2 ± 9.7 ^c^
	M5	150.1 ± 7.7 ^d^					27.7 ± 0.4 ^c^	12.6 ± 0.2 ^c^				21.6 ± 0.1 ^c^	1 ± 0.0 ^d^	15.5 ± 2.8 ^c^	8.4 ± 1.0 ^c^	236.9 ± 4.1 ^d^

Note: Values indicate Average ± Standard deviation. Lowercase letters next to the deviation indicate significant differences with *p* < 0.05, between different degrees of ripeness for the same variety. Several cells indicate the absence of the compound.

**Table 5:** Average values for the minimum inhibitory concentration of dry ethanolic extracts of the pepper varieties under study.

			Bacterial Strain				Fungal Strain	
		Concentration (mg/mL)	*E. coli* ATCC 8739	*S. aureus* ATCC 6538P	*P. aeruginosa* ATCC 9027	*S. mutans* ATCC 25175	*C. albicans* ATCC 1031	*C. tropicalis* ATCC 13803
Malagueta chili	M1	151.8	-	63.2	-	-	-	-
	M2	156.1	-	-	-	16.3	-	-
	M3	200.1	-	-	-	-	-	-
	M4	150.9	15.7	62.9	-	62.9	-	-
	M5	213.0	-		-	-	-	-
Habanero chili	M1	163.5	17.0	8.5	-	8.5	-	-
	M2	154.2	4.0	-	-	16.1	-	-
	M3	200.3	-	20.9	-	-	-	-
	M4	93.0	9.7	-	-	-	-	-
	M5	168.4	17.5	70.2	-	4.4	-	-
Jalapeño chili	M1	152.4	7.9	-	63.5	2.0	-	-
	M2	153.8	16.0	-	64.1	2.0	-	-
	M3	156.3	16.3	65.1	65.1	2.0	-	-
	M4	150.5	15.7	-	62.7	7.8	-	-
	M5	155.7	16.2	-	-	2.0	-	-
Orange cherry peppers	M1	100.0	-	-	41.7	41.7	-	-
	M2	100.0	-	-	-	-	-	-
	M3	100.0	41.7	41.7	-	-	41.7	41.7
	M4	100.0	-	-	41.7	-	-	-
	M5	100.0	-	-	20.8	20.8	-	-
Yellow medium peppers	M1	100.8	10.5	21.0	-	10.5	10.5	-
	M2	100.0	41.7	-	-	-	-	-
	M3	100.0	10.4	41.7	-	-	-	-
	M4	200.0	2.6	41.7	-	-	-	-
	M5	100.0	20.8	41.7	-	20.8	-	-
Orange medium peppers	M1	100.2	10.4	-	-	41.7	20.9	20.9
	M2	100.0	10.4	-	-	2.6	41.7	-
	M3	200.0	5.2	10.4	-	-	-	-
	M4	200.0	5.2	5.2	-	20.8	-	-
	M5	100.5	10.5	-	-	10.5	-	-
Yellow big peppers	M1	100.0	-	-	-	-	-	-
	M2	100.0	41.7	-	41.7	20.8	41.7	41.7
	M3	100.2	41.7	-	41.7	20.8	-	-
	M4	100.0	10.4	-	41.7	41.7	-	-
	M5	100.0	10.4	-	41.7	41.7	-	-

Note: -, non-active at the tested concentration.

## Data Availability

The original contributions presented in this study are included in the article. Further inquiries can be directed to the corresponding author.
